# School distress and the school attendance crisis: a story dominated by neurodivergence and unmet need

**DOI:** 10.3389/fpsyt.2023.1237052

**Published:** 2023-09-22

**Authors:** Sophie E. Connolly, Hannah L. Constable, Sinéad L. Mullally

**Affiliations:** ^1^Translational and Clinical Research Institute, Faculty of Medical Sciences, Newcastle University, Newcastle upon Tyne, United Kingdom; ^2^School of Psychology, Newcastle University, Newcastle upon Tyne, United Kingdom

**Keywords:** school distress, school refusal, school attendance difficulties, autism, pathological demand avoidance, sensory processing disorder, mental health, ADHD

## Abstract

**Background:**

The Covid-19 pandemic has brought into sharp focus a school attendance crisis in many countries, although this likely pre-dates the pandemic. Children and young people (CYP) struggling to attend school often display extreme emotional distress before/during/after school. We term this School Distress. Here we sought to elucidate the characteristics of the CYP struggling to attend school in the United Kingdom.

**Methods:**

Using a case–control, concurrent embedded mixed-method research design, 947 parents of CYP with experience of School Distress completed a bespoke online questionnaire (February/March 2022), alongside an age-matched control group (*n* = 149) and a smaller group of parents who electively home-educate (*n* = 25).

**Results:**

In 94.3% of cases, school attendance problems were underpinned by significant emotional distress, with often harrowing accounts of this distress provided by parents. While the mean age of the CYP in this sample was 11.6 years (StDev 3.1 years), their School Distress was evident to parents from a much younger age (7.9 years). Notably, 92.1% of CYP currently experiencing School Distress were described as neurodivergent (ND) and 83.4% as autistic. The Odds Ratio of autistic CYP experiencing School Distress was 46.61 [95% CI (24.67, 88.07)]. Autistic CYP displayed School Distress at a significantly earlier age, and it was significantly more enduring. Multi-modal sensory processing difficulties and ADHD (among other neurodivergent conditions) were also commonly associated with School Distress; with School Distress CYP having an average of 3.62 NDs (StDev 2.68). In addition, clinically significant anxiety symptomology (92.5%) and elevated demand avoidance were also pervasive. Mental health difficulties in the absence of a neurodivergent profile were, however, relatively rare (6.17%). Concerningly, despite the striking levels of emotional distress and disability reported by parents, parents also reported a dearth of meaningful support for their CYP at school.

**Conclusion:**

While not a story of exclusivity relating solely to autism, School Distress is a story dominated by complex neurodivergence and a seemingly systemic failure to meet the needs of these CYP. Given the disproportionate number of disabled CYP impacted, we ask whether the United Kingdom is upholding its responsibility to ensure the “right to an education” for all CYP (Human Rights Act 1998).

## 1. Introduction

“*A withered boy who was so afraid, hiding from society in the shade, His solitary cries no-one did hear, his confused mind full of fear. His tortured soul locked inside, with his faded dreams that had died.”* Damian Milton, Autistic scholar (1).

School attendance problems driven by mental health (MH) difficulties are increasingly prevalent in the United Kingdom with the Children’s Commissioner’s recent Attendance Audit [“Where are England’s Children?,” (2)] estimating that 1.7 million pupils in England were persistently absent (missing over 10% of school sessions) and 124,000 pupils severely absent (missing over 50% of school sessions) in the autumn 2021 term.

School attendance problems underpinned by MH difficulties are, however, not a new phenomenon. Failure by the scientific community to agree a typology for describing attendance problems driven by MH difficulties (3) has hindered understanding and support, and led to a phenomena that is poorly described in the literature (4). Terms such as “school refusal,” “school phobia” and “school avoidance” have been used interchangeably throughout the literature to describe attendance problems, with “school refusal behavior” frequently used as an umbrella term covering anxiety-based school refusal and truancy (3).

**Figure 1:**
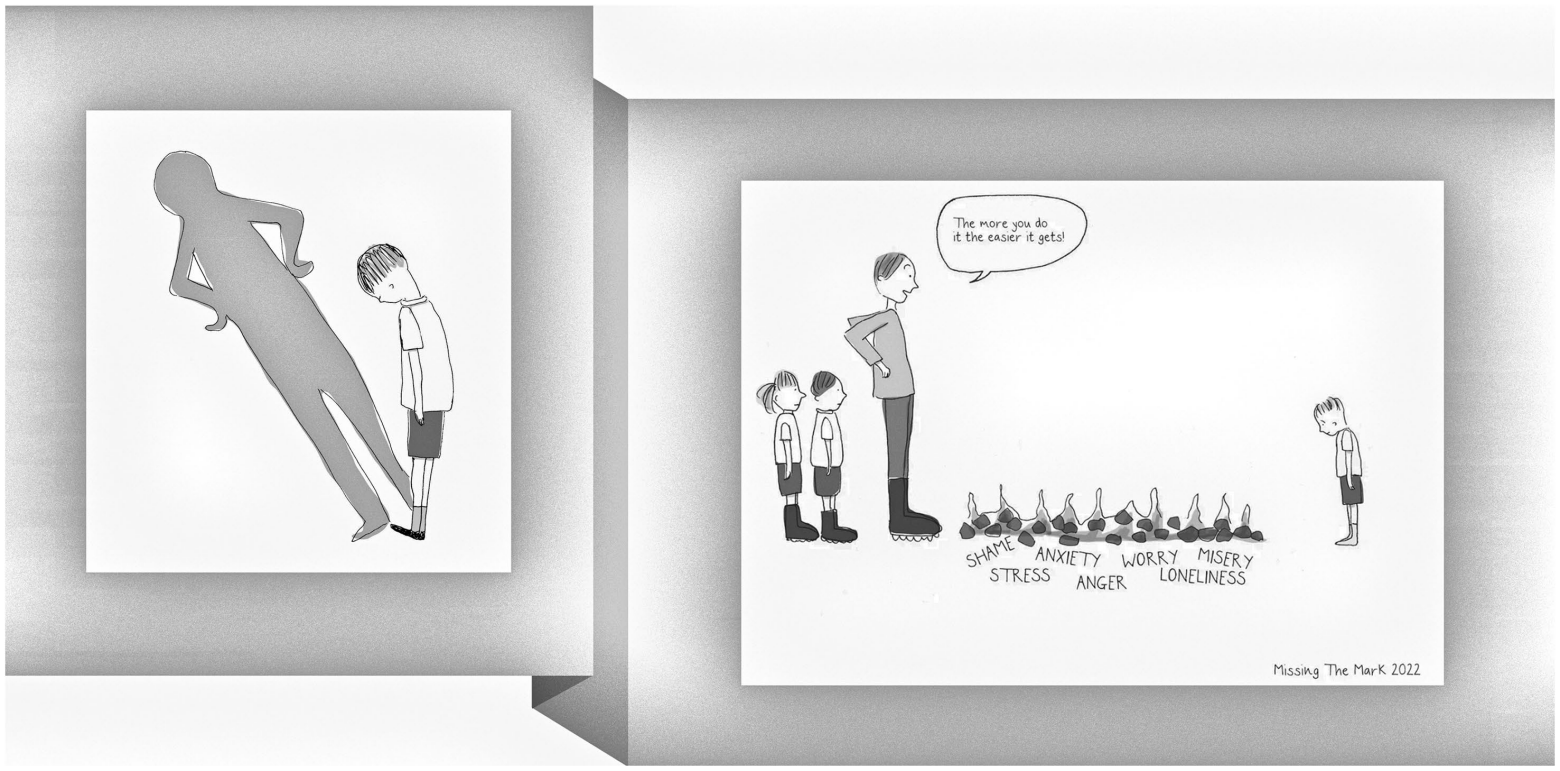
Visual illustrations depicting the experience of School Distress from a young child’s perspective. The two illustrations capture aspects of both the child’s experience of being unable to (as opposed to ‘refusing’ to) attend school and common adult responses to this presentation. Reproduced with permission from the author and illustrator Eliza Fricker (https://missingthemark.blog/).

Terms such as school “refusal” and “avoidance” are, however, rejected by many individuals with lived experience of school attendance problems driven by MH difficulties, as they suggest the behavior is under the control of the young person (5–10). Moreover, they do not convey any information regarding the emotional distress associated with school attendance experienced by these children and young people (CYP) (6, 7, 11–13) (see Figure 1). The term “School Anxiety,” which first appeared in the literature in 1959 (14), has become increasingly prevalent over recent years, as it goes some way in addressing the above concerns. However, it is narrow, focusing only on the anxiety component of the CYP’s experience. This places the focus on treating the CYP’s anxiety, as opposed to simultaneously addressing the drivers of this anxiety (6, 7, 11, 12).

We propose that attendance problems underpinned by emotional distress are best described as “School Distress” (SD), given that emotional distress associated with school attendance is the core driving feature (see Figure 1). This term does not focus solely on the anxiety component of the phenomena, which may be the outcome, rather than the driver, of the distress experienced by the CYP at school. Unlike terms such as “School Refusal,” School Distress is person-orientated and, as such, attempts to convey information to individuals surrounding CYP with respect to the child’s experience and presentation. We hope that this will intrinsically foster greater understanding and earlier recognition (particularly to early signs of distress), ultimately leading to more empathetic, appropriate support for these CYP.

Historically school attendance problems are considered equally common among boys and girls, with no noted socioeconomic component (15). The onset of attendance problems may be sudden or gradual, with possible presentations including children pleading to miss school, displaying physical refusal in the morning, or expressing somatic complaints (16, 17).

Although School Distress likely accounts for a significant proportion of school absences, official figures are not available in the United Kingdom, with absences due to MH difficulties not being recorded differently to general absences (18). This prevents a full estimation of the scale of the problem. However, even if available, these figures would exclude CYP experiencing School Distress who still manage to attend school but who experience significant distress while there. Some authors estimate that attendance problems due to emotional distress affect around 1% of school-aged children (19), although others suggest higher estimates [e.g., (15, 20)], likely due to the different conceptualizations used (21).

Within current literature, limited research has directly explored the causes of School Distress. Despite this, some potential factors have emerged within small-scale interviews with CYP and their parents, including fear of teacher behavior, noisy and disorganized classrooms, anxiety, isolation, and unpredictability (22–24). In recent years, autism and sensory processing difficulties have become increasingly recognized as common characteristics among CYP experiencing school attendance problems (25).

### 1.1. Autism

Autism impacts how individuals makes sense of the world around them. These differences often result in communication breakdowns between autistic and non-autistic individuals, likely due to the differing perspectives between the two neurotypes (26, 27). Significant differences in the primary sensory experiences of the autistic lived experience are widely documented [e.g., (28)], with previous estimates of sensory processing differences in ~90% of autistic individuals (29, 30). Elevated rates of anxiety are also pervasive (31, 32).

Strikingly, Ochi et al. (33) found 40% of their school ‘refusing’ participants to be autistic, while Munkhaugen et al. (34) identified teacher-reported attendance problems in 42.6% of autistic students, compared to 7.1% of neurotypical (NT) students. Munkhaugen et al. (34) also reported that these differences persisted when primary and secondary students were studied separately, and that autistic CYP were absent on significantly more days than their neurotypical peers, indicating greater severity. This aligns with Ochi et al. (33) who reported a significantly lower age of onset of attendance problems in autistic children.

Insights into why autistic CYP have disproportionately negative experiences at school, and thus why they appear to be at increased risk of School Distress, are available from multiple sources (35–39). Contributing factors overlap with previously identified drivers (22–24) and include sensory processing difficulties, feelings of exclusion, lack of teacher understanding, anxiety and demand avoidance.

### 1.2. Sensory processing difficulties

There is increasing evidence that sensory processing difficulties affect CYPs’ school experiences (38), with mainstream school environments often consisting of “sensory exclusion” that disadvantage and marginalize autistic CYP (40). In support of this, parents in Havik et al.’s study (23) highlighted noisy classrooms as a contributing factor to School Distress, and Dougal et al. (41) reported that teachers with experience teaching autistic children in mainstream and SEN provisions identified sensory issues as a key barrier to learning in classroom settings. Furthermore, Jones et al. (36) found that negative sensory experiences in school can impact learning, cause distraction and anxiety, and limit participation in education.

The high prevalence of sensory processing differences in autistic CYP (29, 30, 42) could explain the increased prevalence of School Distress among autistic CYP. However, non-autistic neurodivergent CYP (such as CYP with ADHD) also experience sensory processing differences (43), as do many other CYP, such as CYP born prematurely (44). Hence, both autistic and non-autistic CYP who experience sensory processing difficulties may be at heightened risk of experiencing School Distress.

### 1.3. Anxiety

Anxiety may also play an important role in the emergence and/or persistence of School Distress for many CYP [e.g., (24)], with high anxiety levels commonly noted in CYP experiencing attendance problems [e.g., (45)]. For example, Jones et al. (46) found significantly greater clinician- and child-reported anxiety severity among school-reluctant CYP, compared to non-school reluctant CYP. Moreover, in a study of Ecuadorian adolescents, Gonzalvez et al. (47) found that CYP whose school ‘refusal’ was strongly linked to avoidance of negative affectivity, escape from aversive social and/or evaluative situations, and/or pursuit of attention, had significantly elevated depression, anxiety and stress. Thus, high anxiety appears to be another characteristic of CYP experiencing School Distress. These studies do not, however, tell us whether high anxiety is a cause or a consequence of CYPs’ distressing experiences in school.

As severe symptoms of anxiety frequently co-occur in autistic individuals (32), understanding the role of both autism and anxiety (and the intersection between the two) in the development and maintenance of School Distress is likely important in elucidating key drivers of this phenomenon. Similarly, other neurodivergent conditions that also frequently co-occur with anxiety may also heighten the risk of experiencing School Distress [e.g., ADHD (48)], as too could a primary diagnosis of anxiety.

### 1.4. Demand avoidance

Parents of CYP experiencing school attendance problems often highlight their child’s difficulties coping with everyday demands as being instrumental in the difficulties faced at school, leading to extreme distress and/or behaviors (6). Such demands are omnipresent in the adult-directed mainstream setting, indicating a potential link between School Distress and demand avoidance.

Pathological Demand Avoidance (PDA) was first described by Newson (49, 50), to describe a group of CYP who displayed seemingly “obsessive resistance” to everyday demands and an extreme need for control (51). Although research is limited, a population cohort study from the Faroe Islands suggested almost 1/5 autistic CYP show some demand avoidant characteristics (52). Demand avoidance in adults may be anxiety-driven (53), and in CYP these behaviors may be an attempt to increase certainty/predictability in order to alleviate increasing anxiety (54). PDA is also described in the literature as ‘Extreme Demand Avoidance’ (EDA) (52), although some advocates suggest ‘Rational Demand Avoidance’ (RDA) [i.e., as a rational response to one’s circumstances (1, 55)] or ‘Pervasive Drive for Autonomy’ (56) as more appropriate typology.

Importantly, a key motivation for Newson’s recognition of PDA was that a lack of recognition of this “*markedly divergent overall presentation...contributes to inappropriate handling and educational methods, since PDA children respond best to very different approaches compared with those suitable for autistic and Asperger children*.” More recently, Summerhill and Collett (57) highlighted anecdotal evidence indicating that when demand avoidant CYP are not identified, their presentation is viewed as defiance and deliberately challenging behavior, leading to school exclusions (58), which negatively impacts access to education, social relationships, and MH (57).

Moreover, the uniqueness of the demand avoidant profile may explain why a 2018 survey found that 70% of school aged demand avoidant CYP were either not enrolled in school or were unable to tolerate their school environment (59). Additionally, Truman et al. (60) found that while all parents of autistic CYP described their child’s school experiences as overwhelmingly negative, parents of demand avoidant autistic CYP provided markedly more negative descriptions than parents of autistic CYP without demand avoidant profiles. Hence, demand avoidant autistic children may be especially vulnerable to School Distress, perhaps due to their elevated levels of anxiety (61) and need for alternative educational methods (49, 50), which likely require a flexible, non-directive teaching style (62), as pressure to comply with direct demands is well-documented to lead to escalation in emotional reactivity and challenging behavior (63).

Interestingly, while the PDA profile is recognized by autism specialist clinicians and academics as an important known range of co-occurring difficulties for many autistic individuals (64), PDA has also been documented in other neurodivergent profiles such as selective mutism, language disorders, epilepsy, and, less commonly, in the general population (52). Understanding how demand avoidance relates to School Distress, and the parameters discussed above (e.g., neurotype, anxiety, sensory processing differences), is thus important to fully elucidate the factors that contribute to School Distress. To date, however, there is a dearth of academic research exploring this link.

### 1.5. Current research

In this research, we sought to address the current dearth of understanding in the literature by addressing a number of outstanding questions. More specifically, by comparing CYP who have experienced School Distress with both CYP who attend school without distress and CYP who have never attended a school setting [i.e., Electively Home-Educated (EHE) CYP], we aimed to 1. identify prevalent characteristics of CYP who have experienced difficulties attending school; 2. quantify the proportion of cases of school attendance problems associated with emotional distress; 3. compare anxiety levels, sensory processing difficulties, and demand avoidance profiles between the three groups; 4. explore associations between sensory processing difficulties, anxiety, demand avoidance, and markers of School Distress severity (i.e., duration of School Distress, school attendance rate, age of onset, and impact of school attendance on MH); and 5. assess the level of support received by CYP currently experiencing School Distress.

It is hypothesized that neurodivergent (ND) CYP will be over-represented among individuals with School Distress experience, particularly autistic CYP and CYP with sensory processing difficulties; that anxiety will be prevalent in CYP with School Distress, particularly in autistic, non-autistic ND, and/or demand avoidant CYP; and CYP with more extensive sensory processing difficulties, higher anxiety, and more pervasive demand avoidant profiles will show more severe School Distress than their neurotypical peers.

## 2. Materials and methods

### 2.1. Participants

**Figure 2:**
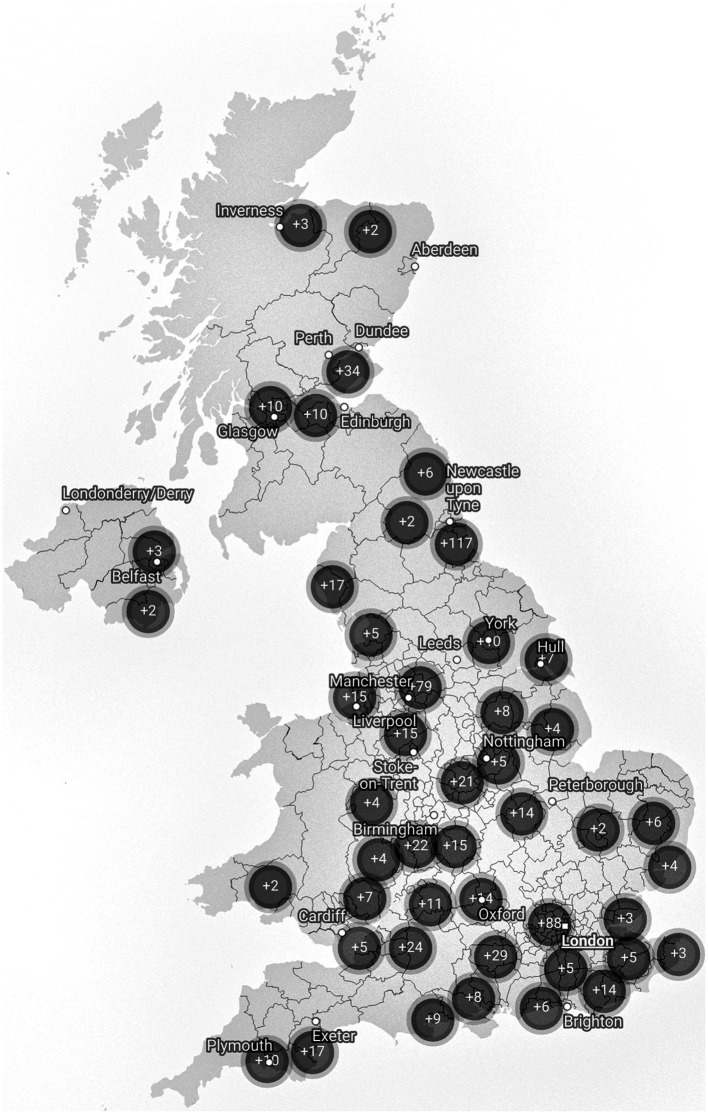
Map of CYP currently experiencing School Distress. Single cases have been removed to ensure anonymity. This is a sample specific distribution and may not reflect the true geographical distribution of people experiencing School Distress. Map data: © Crown copyright and database right 2021. Created with DataWrapper.

Participants were required to live in the United Kingdom and be parents/carers of school-aged CYP. Initially, 1,055 participants were recruited via volunteer sampling, consisting of 738 parents of children currently experiencing School Distress (Current SD), 209 parents of children who have previously experienced School Distress (Past SD), 83 parents of children who have never experienced School Distress (No SD), and 25 parents of children who have never attended a school setting (Lifelong EHE). An additional 66 parents of CYP who have never experienced School Distress were recruited via prolific.org to ensure the Current, Past, and No School Distress groups were all matched in terms of chronological age, providing an overall sample of 1,121 participants. To assist with age matching, prolific parents with more than one child were instructed to consider their eldest child within the questionnaire. On average, participants completed 77.35% of the survey, with 62.5% completing 100%. Most participants were mothers (97.03%). Table 1 displays key characteristics of the CYP. Figure 2 shows a map of the CYP experiencing School Distress.

**Table 1:** Demographic characteristics of the sample.

Characteristic	All	Current SD	Past SD	No SD	Lifelong EHE
*n* (%)	1,121	738 (65.8)	209 (18.6)	149 (13.3)	25 (2.2)
Mean Age in Years ± StDev	11.6 ± 3.3	11.8 ± 3.1	11.8 ± 3.6	11.1 ± 3.5	8.7 ± 3.0
IMD decile	6.04 (2.9)	6.16 (2.8)	5.51 (3.1)	6.17 (2.9)	5.50 (2.6)
Respondent’s relationship to child (%)					
Mother	1,077 (97.0)	707 (96.6)	200 (98.0)	145 (97.3)	25 (100)
Father	18 (1.6)	14 (1.9)	1 (0.5)	3 (2.0)	0
Other	15 (1.4)	11 (1.5)	3 (1.5)	1 (0.7)	0
Gender (%)					
Cisgender boy	577 (52.1)	381 (52.1)	117 (57.6)	68 (45.6)	11 (44.0)
Cisgender girl	471 (42.5)	300 (41.0)	77 (37.9)	80 (53.7)	14 (56.0)
Transgender boy	9 (0.8)	9 (1.2)	0	0	0
Transgender girl	1 (0.1)	0	1 (0.5)	0	0
Non-binary	27 (2.4)	23 (3.1)	4 (2.0)	0	0
Self-describe	11 (1.0)	8 (1.1)	3 (1.5)	0	0
Prefer not to say	12 (1.1)	10 (1.4)	1 (0.5)	1 (0.7)	0
Ethnic group (%)					
White	693 (93.4)	458 (93.5)	113 (95.8)	106 (90.6)	16 (94.1)
Mixed/Multiple Ethnic Groups	37 (5.0)	24 (4.9)	4 (3.4)	9 (7.7)	0
Asian/Asian British	6 (0.8)	3 (0.6)	1 (0.8)	1 (0.9)	1 (5.9)
Black/African/Caribbean/Black British	3 (0.4)	2 (0.4)	0	1 (0.9)	0
Other ethnic group	3 (0.4)	3 (0.6)	0	0	0
Main language (%)					
English	735 (99.3)	484 (99.2)	117 (100)	117 (99.2)	17 (100)
Other	5 (0.7)	4 (0.8)	0	1 (0.8)	0
Country of residence (%)					
England	980 (88.3)	644 (88.0)	183 (89.7)	129 (86.6)	24 (96.0)
Scotland	94 (8.5)	68 (9.3)	13 (6.4)	13 (8.7)	0
Wales	22 (2.0)	14 (1.9)	5 (2.5)	2 (1.3)	1 (4.0)
Northern Ireland	14 (1.3)	6 (0.8)	3 (1.5)	5 (3.4)	0
Siblings (%)					
Yes	850 (79.7)	571 (81.1)	145 (75.9)	118 (79.2)	16 (69.6)
No	217 (20.3)	133 (18.9)	46 (24.1)	31 (20.8)	7 (30.4)
Position in family (%)					
Youngest	329 (33.6)	250 (39.2)	54 (30.5)	20 (14.1)	5 (22.7)
Middle	92 (9.4)	68 (10.7)	17 (9.6)	5 (3.5)	2 (9.1)
Eldest	314 (32.1)	169 (26.5)	57 (32.2)	81 (57.0)	7 (31.8)
Twin	27 (2.8)	18 (2.8)	3 (1.7)	5 (3.5)	1 (4.5)
Only child	217 (22.2)	133 (20.8)	46 (26.0)	31 (21.8)	7 (31.8)
SEN Support (*Mainstream only)					
No SEN support*	-	152 (35.9)	36 (100)	122 (97.6)	n/a
SEN support*	-	194 (45.5)	16 (100)	15 (10.6)	n/a
EHCP/Statement in place*	-	91 (21.2)	19 (26.4)	6 (4.2)	n/a
EHCP/Statement in progress*	-	64 (14.9)	0	0	n/a

SD, School Distress; EHE, Electively Home Educated; SEN, Special Educational Needs; EHCP, Education, Health and Care Plan.

### 2.2. Language

Where possible, we use identity-first language (e.g., autistic CYP) (65). We defined neurodivergence (ND) for parents as “a term for when someone’s brain processes, learns and behaves differently from what is considered ‘typical’. Autism is an example of a neurodivergence.” We use the term ‘non-autistic’ to refer to CYP whose parents did not identify them as autistic (be that diagnosed or self/parent-identified), and ‘non-autistic ND CYP’ for the subgroup of non-autistic CYP who are otherwise neurodivergent. We use the term ‘neurotypical’ (NT) to refer to CYP whose parent identified them as not being neurodivergent.

### 2.3. Design

The study employed a case–control, concurrent embedded, mixed-methods design where qualitative data was collected to supplement quantitative data. This was chosen due to the study’s exploratory nature, and because the limited literature prevented us from providing fully comprehensive lists of response options to some questions. To collect qualitative data, text boxes were presented within some questions for parents to provide comments. The results reported in this paper are largely quantitative, with some parental comments reported to support understanding. Thematic analyzes and additional data will be reported elsewhere.

### 2.4. Materials

A bespoke online questionnaire was developed containing four sections and 76 questions. Only certain questions were presented to each respondent, based on their experience of School Distress and survey responses. Questions and response options were developed based upon a comprehensive literature review, and aimed to collate key information about the respondent, their CYP, their CYP’s experience of School Distress, and the impact of School Distress on themselves.

This paper will report data from the questions and clinical scales described below. Data relating to how School Distress presents, the reasons underlying School Distress, the efficacy of supports, the consequences of School Distress, and the parental experience will be reported elsewhere.

(1) Demographic information: Participants were asked their relationship to the CYP, and the CYP’s country of residence, spoken language, ethnicity, age, gender identity, and number of siblings (with ages). Postcodes were requested and converted into Index of Multiple Deprivation (IMD) Deciles for families living in England (66). In accordance with prolific.org data protection policy, prolific participants provided only their IMD decile.^1^
(2) School attendance problems: Participants were asked whether their child has ever experienced difficulties attending school (response options: ‘currently’, ‘in the past’, ‘never’, or ‘not applicable as child never attended a school setting’), and if so, what age their difficulties began. Where attendance difficulties were current, parents were asked how long they had been ongoing, and how many days their CYP had attended school over the proceeding 20 school days. Attendance rates for the current (2021/22) and previous academic year (2020/21; excluding Covid-19-related absences) were also estimated. All parents of CYP with school attendance problems were asked to describe these difficulties using one of the following options: “Self-corrective school avoidance (i.e., absenteeism that remits spontaneously within 2 weeks)”; “Acute school avoidance (i.e., absenteeism that lasts from 2 weeks to 1 year)”; “Chronic school avoidance (i.e., absenteeism that lasts longer than 1 year)”; “None of the above. It looks more like...(please describe).” In addition, all parents were asked about the impact of attending school on their child’s MH [response options: ‘Extremely positively’ (+3), ‘Very positively’ (+2), ‘Somewhat positively’ (+1), ‘Neither positively nor negatively’ (0), ‘Somewhat negatively’ (−1), ‘Very negatively’ (−2), ‘Extremely negatively’ (−3)]. Detailed results will be presented elsewhere, however this measure will be used here as a marker of School Distress severity. Finally, parents were also asked whether the CYP’s siblings have a history of school attendance problems.
(3) Educational information: Parents were asked to indicate the types of educational provision their child currently (and if relevant, previously) attended, the total number of schools their child had attended, and whether their child was currently receiving SEN support at school [response options: ‘receives no additional support’, ‘receives SEN support (e.g., is on the SEN register)’, or ‘has an EHCP/Statement/CSP/ALN (or similar) in place or in process’].
(4) Child health and neurodivergencies: To better understand the needs of CYP struggling to attend school, parents were asked if their child has any physical or MH difficulties (and, if so, what they were), and if they are neurodivergent. Parents who stated their child was (or might be) neurodivergent were provided with a list of possible neurodivergencies (see Table 2) and asked to select all that apply to their child (response options: ‘has a clinical diagnosis’, ‘is on the diagnostic pathway’, ‘had a referral refused’, ‘suspected but has never been referred and/or diagnosed’). Unless otherwise indicated, prevalence rates for each neurodivergent condition were calculated by accepting endorsement of any of these four options.
  Parents who identified their CYP to have sensory processing disorder were presented with the eight sensory systems (visual, auditory, tactile, olfactory, gustation, vestibular, proprioceptive, and interoceptive) and asked to identify those in which their CYP experienced difficulties. Parents who selected intellectual disability were asked if this was best described as ‘mild–moderate’, ‘severe’, or ‘profound’. To gain a wider understanding of the CYP’s family history, we asked whether either of the CYP’s parents, or their siblings, are neurodivergent. Rates of parental neurodivergence will be published elsewhere.
  All parents were asked to complete the 24-item Anxiety Scale for Children–Autism Spectrum Disorder–Parent Version (ASC-ASD-P) (67), which was derived from a well-validated measure used with typically-developing children (68) and developed for use with autistic CYP. The ASC-ASD-P was selected given the anticipated rates of autistic CYP in our sample. This parent-report measure provides a total anxiety score, and individual scores for Separation Anxiety, Uncertainty, Performance Anxiety, and Anxious Arousal (total anxiety will be presented here, and findings regarding specific subscales will be reported elsewhere). Parents respond along a 4-point Likert scale (0 = Never, 1 = Sometimes, 2 = Often, 3 = Always), and Finally, we explored scores are calculated by summing responses, with higher scores indicating greater levels of anxiety (range 0–72). A total score of 20–23 suggests “significant anxious symptomatology,” and scores above 24 suggest a “more specific indication of significant anxiety” (67). The ASC-ASD-P has excellent internal consistency (α = 0.94) and good convergent validity.
(5) Demand avoidance: All parents were asked to complete the 8-item Extreme Demand Avoidance-8 Caregiver Report Questionnaire (EDA-8) (69), which is a refined version of the Extreme Demand Avoidance Questionnaire (EDA-Q) (70). The scale’s items cover features consistently described in accounts of EDA: obsessive avoidance of demands and requests, outrageous or shocking behavior to avoid, need for control, poor awareness of hierarchy, and lability of mood. The EDA-8 has good internal consistency (*α* = 0.90) and convergent and divergent validity, and is proposed to be a useful tool to identify children showing an extreme response to demands (69). Parents respond along a 4-point Likert scale (0 = Not at all true, 1 = Somewhat true, 2 = Mostly true 3 = Very true). Scores are calculated by summing responses, with higher scores indicating greater EDA. Cut-off scores are not currently available.

**Table 2:** Frequency (%) of individual neurodivergencies in each group, and average number of neurodivergencies per group.

Neurodivergence	Current SD	Past SD	No SD	Lifelong EHE	Between-group analysis
Neurodivergent (ND)	666 (92.1)	168 (83.6)	33 (22.2)	22 (88.0)	*χ*^2^(3,1,098) = 394.5, *p* < 0.001
Autistic	598 (83.4)	133 (66.2)	25 (16.8)	13 (52.0)	*χ*^2^(3,1,092) = 269.7, *p* < 0.001
Sensory Processing Disorder/Sensory Integration Disorder (SPD/SID)	406 (56.9)	87 (43.3)	10 (6.7)	13 (52.0)	*χ*^2^(3,1,089) = 126.2, *p* < 0.001
Attention Deficit Hyperactivity Disorder (ADHD)	395 (55.3)	87 (43.3)	13 (8.7)	12 (48.0)	*χ*^2^(3,1,089) = 108.6, *p* < 0.001
Dyslexia	181 (25.4)	47 (23.4)	8 (5.4)	2 (8.0)	*χ*^2^(3,1,089) = 31.9, *p* < 0.001
Dyspraxia	177 (24.7)	55 (27.4)	12 (8.1)	2 (8.0)	*χ*^2^(3,1,089) = 108.6, *p* < 0.001
Auditory Processing Disorder (APD)	130 (18.2)	26 (12.9)	5 (3.4)	3 (12.0)	*χ*^2^(3,1,089) = 22.4, *p* < 0.001
Speech Difficulties	115 (16.1)	21 (10.4)	7 (4.7)	4 (16.0)	*χ*^2^(3,1,089) = 15.8, *p* < 0.01
Gifted	103 (14.4)	29 (14.4)	6 (4.0)	6 (24.0)	*χ*^2^(3,1,089) = 14.7, *p* < 0.01
Other	89 (12.5)	16 (8.0)	3 (2.0)	3 (12.0)	*χ*^2^(3,1,089) = 16.1, *p* < 0.01
Dyscalculia	88 (12.3)	19 (9.5)	4 (2.7)	0	*χ*^2^(3,1,089) = 15.7, *p* < 0.01
Language Disorder	82 (11.5)	20 (10.0)	4 (2.7)	2 (8.0)	*χ*^2^(3,1,089) = 10.8, *p* < 0.05
Visual Processing Difficulties	69 (9.7)	19 (9.5)	0	0	*χ*^2^(3,1,089) = 18.2, *p* < 0.001
Tic Disorder	61 (8.5)	13 (6.5)	5 (3.4)	1 (4.0)	*χ*^2^(3,1,089) = 5.6, *p* > 0.05
Unspecified Learning Disorder	60 (8.4)	10 (5.0)	1 (0.7)	1 (4.0)	*χ*^2^(3,1,089) = 13.4, *p* < 0.01
Intellectual Disability	48 (6.7)	10 (5.0)	2 (1.3)	1 (4.0)	*χ*^2^(3,1,089) = 7.1, *p* > 0.05
Spatial Processing Disorder	46 (6.4)	10 (5.0)	2 (1.3)	0	*χ*^2^(3,1,089) = 7.9, *p* < 0.05
Mean Number of NDs (StDev)	3.7 (2.5)	3 (2.5)	0.72 (1.7)	2.5 (2.0)	*F*(3,1,085) = 61.0, *p* < 0.001

Frequencies reflect the number of parents who responded “yes” or “maybe” to their child being neurodivergent and having each neurodivergent condition. Under the “other” category, hypermobility, PDA, and dysgraphia were the most frequently mentioned. Chi-Square (*χ*^2^) analyzes were used to explore between-group differences for categorical variables, while the quantitative variable (i.e., number of ND conditions per CYP) was explored using a one-way ANOVA.

### 2.5. Procedure

Data was collected using Qualtrics. The survey link was shared widely on social media, and the additional control participants recruited via prolific.org were directed to the Qualtrics link. The original advertisement for the study was posted on Facebook. The original post was ‘liked’ 441 times, had 60,944 Post Impressions, 48,649 Post Reaches, and 3,714 Post Engagements. Organizations that shared the advertisement included parent support groups such as ‘Not Fine in School–Public Page–School Attendance Difficulties’ (~29,000 members) and ‘Define Fine: Parent Peer Support for School Attendance’ (~6,200 members; see Supplementary material for more details).Participants read the information sheet and provided consent before beginning the survey. They were informed that they could skip any questions and stop/start at any time. Qualtrics’ display-logic function ensured respondents were only asked questions which were relevant to them. Upon completion, participants were presented with a debrief form, including a comprehensive list of support services. The study ran for 14 days (22/02/2022–08/03/2022).

### 2.6. Data analysis

Participants were designated to one of four groups based upon whether their child had ever experienced difficulties attending school: the response option ‘Yes, currently’ assigned them to the Current School Distress group, ‘Not currently, but they have in the past’ to the Past School Distress group, ‘No, never’ to the No School Distress group, and ‘Not applicable as child never attended a school setting’ to the Lifelong EHE group.

Quantitative data analyzes were run using IBM SPSS Statistics V26. Descriptive statistics were calculated to summarize participants’ responses to each question. Further statistical analyzes were conducted to examine relationships between variables. Before performing statistical analyzes, normality was assessed by plotting results in histograms and conducting Shapiro–Wilk and Kolmogorov–Smirnov tests. When results were not normally distributed, non-parametric methods were used. A significance level of *α* = 0.05 was adopted for most analyzes, and a Bonferroni-corrected alpha level was used to correct for multiple comparisons. Odds Ratios (ORs) were calculated as an estimate of effect size. Correlational analyzes were conducted to examine the relationships between variables using Spearman Rho correlations.

As CYP in the Lifelong EHE group were significantly younger than CYP in the other groups [(Current SD=Past SD=No SD) > Elective EHE, *p* < 0.001], it was necessary to conduct additional analyzes using more precisely age-matched comparison groups. Hence, for each CYP in the Lifelong EHE group, two age-matched participants were identified from each of the three other groups. The selected CYP from each group were the two CYP closest in age to the corresponding Lifelong EHE CYP. Analyzes were then replicated using this reduced sample, and conclusions specific to the Lifelong EHE group were derived from these results.

Within our study, we measured four key proxy markers of School Distress: duration, age of onset, attendance rates (in the previous 20 school days, 21/22 academic year, 20/21 academic year), and impact of school attendance on MH. Within this paper, we explored correlations between the four proxy markers and several characteristics of our sample (i.e., anxiety, EDA, and the number of sensory systems that CYP experience difficulties in) to see how they related to School Distress severity.

## 3. Results

### 3.1. Demographic information

#### 3.1.1. Gender

52.1% of CYP were cisgender boys, 42.5% cisgender girls, and the remaining 5.4% were split between the non-binary, transgender boy, transgender girl, self-describe, and prefer not to say options.

#### 3.1.2. Age

The mean age of the CYP was 11.6 years. There was an overall between-group difference in age [*F*(3, 1,106) = 8.548, *p* < 0.001], driven by the significantly younger age of the Lifelong EHE CYP [(Current SD=Past SD=No SD) > Lifelong EHE]. No differences remained when considering the Lifelong EHE age-matched subgroups [*F*(3, 171) = 0.084, *p* = 0.969].

#### 3.1.3. Indices of deprivation

IMD Decile data was available for 47% of Current School Distress CYP, 53% of Past School Distress CYP, 59% of No School Distress CYP, and 16% of Lifelong EHE CYP. There were no significant between-group differences in IMD Decile scores [*F*(3,533) = 1.413; *p* = 0.238].

A one sample t-test compared the mean IMD Decile against the population mean, estimated to be 5.5. The overall mean in our sample was 6.04 (StDev = 2.9), which was significantly higher than the population mean, *t*(536) = 4.312, *p* < 0.001, indicating less deprivation. Broken down into groups, the Current (6.16, StDev = 2.82) and No School Distress group means (6.17, StDev = 2.96) were significantly higher than the population mean [Current SD: *t*(347) = 4.368, *p* < 0.001, No SD: *t*(71)=2.127, *p* < 0.001], while the Past School Distress (5.50, StDev = 3.09) and Lifelong EHE group means (5.50, StDev = 2.65) were not significantly different than the population mean [Past SD: *t*(72)=0.16, *p* = 0.987, Lifelong EHE: *t*(3)=0.000, *p* = 1.000].

#### 3.1.4. Birth order

47.3% of Current School Distress CYP were first-born children (26.5% ‘eldest’ children and 20.8% an ‘only’ child), relative to 49.9% who were younger siblings (39.2% ‘youngest’ and 10.7% ‘middle’ children). When considering CYP who had experience of School Distress and who were either the ‘youngest’ or a ‘middle’ child in their family, we found having an older sibling who had also experienced School Distress was common. More specifically, 42.9% of younger siblings in the Current School Distress group, and 46.5% of younger siblings in the Past School Distress group, also had an older sibling/s with a history of School Distress. Similar figures were obtained when ‘youngest’ children were considered in isolation (see Supplementary Figure S1). For a complete description of demographic information, see Table 1.

### 3.2. School attendance difficulties

#### 3.2.1. Age of onset and duration

**Figure 3:**
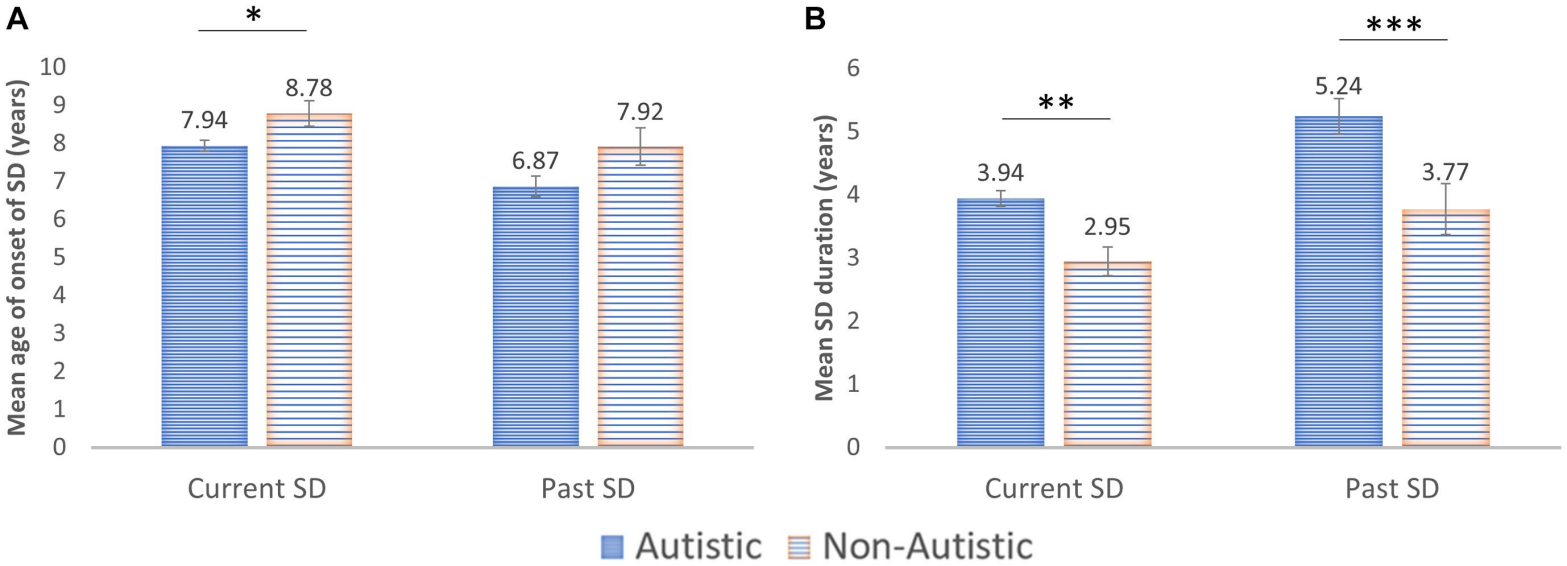
**(A)** Age of Onset of School Distress (SD) in both the Current and Past School Distress groups. **(B)** Duration of School Distress. Error bars: ±1 SEM; *p < 0.05; **p < 0.01; ***p < 0.001.

The mean age of onset of School Distress across both groups was 7.89 years (StDev = 3.37). This was younger in Past School Distress CYP (7.19 years; StDev = 3.21) than Current School Distress CYP (8.07 years; StDev = 3.21). Strikingly, 51.2% of cases of School Distress first occurred at 8 years or younger. The mean duration of School Distress was 3.99 years (StDev = 2.95). This was longer for CYP whose difficulties had now resolved (4.79 years; StDev = 3.12) than for those whose difficulties were still ongoing (3.79 years; StDev = 2.88). Hence, School Distress began significantly earlier in Past School Distress CYP (*p* < 0.01), and lasted significantly longer (*p* < 0.001), likely because the Current School Distress CYP were still experiencing School Distress. The age of onset of School Distress was significantly younger for autistic CYP than non-autistic CYP, and School Distress was reported as being significantly more enduring for autistic CYP (see Figure 3).

Given the timing of this research, with survey completion occurring almost 2 years after the initial Covid-19 school closures began in the United Kingdom, we explored the percentage of School Distress cases that began before and after the Covid-19 pandemic. In most cases, the onset of School Distress preceded Covid-19 related school closures (Current SD: 69.82%; Past SD: 85.15%).

#### 3.2.2. School attendance problems

For 94.32% of CYP, parents indicated their child’s school attendance problems were either partially or fully emotionally-based (i.e., SD).

The existing categories for school “refusal” within the literature (58) (self-corrective, acute, and chronic) failed to capture a significant proportion of the experiences of CYP in this sample (see Supplementary Table S1; Other), with 37.1% of cases (*n* = 320) falling outside of these categories. Examining the Past School Distress group alone, the ‘none of the above’ category was selected by 54.2% of parents. Parents who selected this option were asked to describe what their child’s School Distress looks like. Example responses can be seen in Table 3, Q1. These descriptions capture the distress element of the lived experience of School Distress, as do the additional quotes with respect to how School Distress presents in their children (Table 3, Q2). A full thematic analysis of this data will be described elsewhere.

**Table 3:** A sample of quotations provided by parents in response to specific questions.

Summary of Question	Example Quotes
Q1. Parental descriptions of their CYP’s school attendance difficulties/situation	“Will go but after huge amounts of upset and panic, odd day of refusal followed by managing to go in with difficulty a day or so later” “We would manage to get him into school but he would beg not to go (heartbreaking) and feel nausea from waking until he got home (which he would not tell anyone)” “He attended school but was very distressed, would cry, did not want to go, found being at school pretty stressful (meltdowns at the end of the day)” “We always got him there - but it was hard on many days”“Occasional days off but having to carry a kicking screaming child into school everyday” “We are able to get her into school most days by carrying her...but if she was bigger...she would be missing school” “Will attend more times than not but frequently late/part of day only and does not go to lessons or if he does will read or do his own writing rather than engaging with curriculum” “It’s lasted 10 years! Coping on and off depending on which environment she was in” “Sporadic - in response to situations and difficulties at school with particular lessons, teachers or students” “Extreme withdrawal and a corresponding lack of expression/engagement”
Q2. Parental descriptions of how their CYP’s difficulties present	“Wakes me in the night crying about school” “Sleep disturbance, tummy aches and bed wetting every night prior to school” “Vomiting and incontinence at home,” “Panic on way to school” “Lashing out at myself and car on way to school and on the way home” “Attempt to run in front of traffic, attempts to eat nuts (nut allergy)” “Self harm after a difficult school day” “Attempted suicide due to unmet needs within school”

### 3.3. Educational information

#### 3.3.1. Type of education setting

Overall, 97% of CYP had previously, or were currently, attending a mainstream school setting (Current SD: 97%, Past SD: 97%, No SD: 99%). Almost all CYP in the No School Distress group were currently attending a mainstream school, while just 58.3% of CYP in the Current School Distress group remained in this setting currently. The current and past educational provisions of CYP with School Distress experience will be described in more detail elsewhere.

CYP in both School Distress groups had attended significantly more school settings than CYP without School Distress [*F*(2,1,007) = 12.986, *p* < 0.001; Current SD > No SD, *p* < 0.001; Past SD > No SD, *p* = 0.009]. More specifically, the average number of schools attended by CYP in the Current School Distress group was 2.36 (StDev = 1.094), relative to 2.22 in the Past School Distress group (StDev = 1.164) and 1.86 in the No School Distress group (StDev = 0.814).

#### 3.3.2. Support at school (SEN/EHCP)

Of the Current School Distress CYP, 32.8% received no support at school, 38.1% were on a SEN register (or equivalent), and 48.5% had an EHCP or were in the process of seeking one. This declined to 32.9% when we removed cases where parental comments indicated an EHCP was not yet in place (*n* = 111). Of those cases, 95 parents indicated they were in the EHCP process [e.g., applying, applied, in the assessment phase, in the draft stage/awaiting a finalized plan, at mediation/appealing...]. Some of the mediation/appeals were taking place following a refusal to assess or to issue an EHCP following assessment, while others were appealing the content of sections B (description of the CYP’s SEN), F (provision required to meet needs), and I (specific placement).

Parental dissatisfaction with the support their child is/was receiving from their school and/or local authority (LA) was clear throughout responses. For instance, many described a lack of support in place (e.g., “Very limited support from school”; see Supplementary Table S2, Q1). Even when parents indicated their child was on the school’s SEN register or had an EHCP, comments continued to indicate a lack of support for many CYP (e.g., “Is on the SEN register however no further support in school,” see Supplementary Table S2, Q2). Application for and implementation of EHCPs was also a particular source of frustration, with comments including: “I’d to self-apply as school delayed and blocked” and “School not following EHCP” (see Supplementary Table S2, Q3).

Occasionally comments reflected a more positive situation (e.g., “My child’s school currently provides reasonable adjustments for my daughter’s needs “, see Supplementary Table S2, Q4), however this only represented a small proportion of parent voices.

Finally, some parental comments reflected the complexity of providing support (e.g., “He can receive support from a learning base but he is masking in school and does not want others to know he has autism so only accesses the base twice per week for 50 min per time”).

### 3.4. Child health and neurodivergences

#### 3.4.1. Child health

When asked whether their child has any physical or MH difficulties, only 7% of parents in the Current School Distress group responded ‘No’, compared to 69.8% who stated ‘Yes’ and 23.3% who stated ‘Maybe’. This differed to the other groups, whereby 23.5% of Past School Distress parents, 79.9% of No School Distress parents, and 56% of Lifelong EHE parents responded ‘No’. When asked to specify details of these health difficulties, some parents listed neurodevelopmental conditions. As we later gathered detailed information about neurodivergent conditions, we excluded such responses from analysis (e.g., autism, ADHD, PDA, and sensory processing differences).

Table 4 summarizes the remaining responses with respect to the CYPs’ mental and physical health difficulties, with anxiety being the most frequently mentioned condition in all groups. Depression, Hypermobility, PTSD/trauma, and Low mood/emotional regulation difficulties were the next most common in the two School Distress groups, with few incidences in the No School Distress and Lifelong EHE groups. Hence, MH as opposed to physical health concerns (except for hypermobility) were the most frequently mentioned health difficulties by parents of children with School Distress experience. This should not be considered an exhaustive list, as some parents did not complete this, and others may not have listed all health concerns.

**Table 4:** Frequency and percentage (%) of children within each group who have a range of physical and mental health difficulties, as listed by parents.

Physical/Mental health difficulty	Current School Distress (*n* = 731)	Past School Distress (*n* = 204)	No School Distress (*n* = 149)	Lifelong EHE (*n* = 25)
Anxiety	339 (46.37%)	63 (30.88%)	15 (10.07%)	5 (20%)
Depression	70 (9.58%)	9 (4.41%)	2 (1.34%)	0
Hypermobility/Ehlers Danlos Syndrome	46 (6.29%)	14 (6.86%)	1 (0.67%)	0
PTSD/Trauma	37 (5.06%)	9 (4.41%)	0	0
Low Mood/Emotion Regulation Difficulties	37 (5.06%)	2 (0.98%)	1 (0.67%)	0
Obsessive Compulsive Disorder	24 (3.28%)	6 (2.94%)	1 (0.67%)	0
Low self esteem	14 (1.92%)	0	0	0
Asthma	12 (1.64%)	2 (0.98%)	1 (0.67%)	0
Genetic/Chromosomal disorder (e.g., Down Syndrome, Fragile X Syndrome)	12 (1.64%)	2 (0.98%)	0	0
Eating Disorder/Difficulties/ARFID	12 (1.64%)	1 (0.49%)	0	0
Self-Harm	12 (1.64%)	0	0	0
Attachment issues/Disorder	10 (1.37%)	3 (1.47%)	0	0
Co-ordination difficulties (including DCD)	10 (1.37%)	1 (0.49%)	0	1 (4%)
Phobias (e.g., Agoraphobia, Emetophobia)	10 (1.37%)	0	0	1 (4%)
Unspecified mental health difficulties	10 (1.37%)	0	0	0
Selective mutism	9 (1.23%)	2 (0.98%)	0	2 (8%)
Suicidal ideation/suicide attempts	9 (1.23%)	1 (0.49%)	0	0
Hearing difficulties	6 (0.82%)	0	0	0
Visual impairment	5 (0.68%)	1 (0.49%)	2 (1.34%)	0
Fine and/or gross motor difficulties/delays	5 (0.68%)	1 (0.49%)	0	0
Bladder/bowel difficulties (e.g., IBS)	5 (0.68%)	2 (0.98%)	2 (1.34%)	0
Allergies	5 (0.68%)	1 (0.49%)	2 (1.34%)	0
Sleep difficulties/disorder	5 (0.68%)	1 (0.49%)	0	0
Epilepsy	4 (0.55%)	1 (0.49%)	0	0
Anger issues	4 (0.55%)	0	1 (0.67%)	0
Acid reflux	3 (0.41%)	1 (0.49%)	0	0
Eczema	3 (0.41%)	0	1 (0.67%)	0

As this was an optional free-text question, this should not be considered an exhaustive list. In addition, this table does not include responses including neurodivergent conditions and sensory processing difficulties as these were quantified more precisely in later questions. AFRID, Avoidant Restrictive Food Intake Disorder; DCD, Developmental co-ordination disorder; IBS, Irritable Bowel Syndrome; OCD, Obsessive Compulsive Disorder; PTSD, Post-Traumatic Stress Disorder.

**Figure 4:**
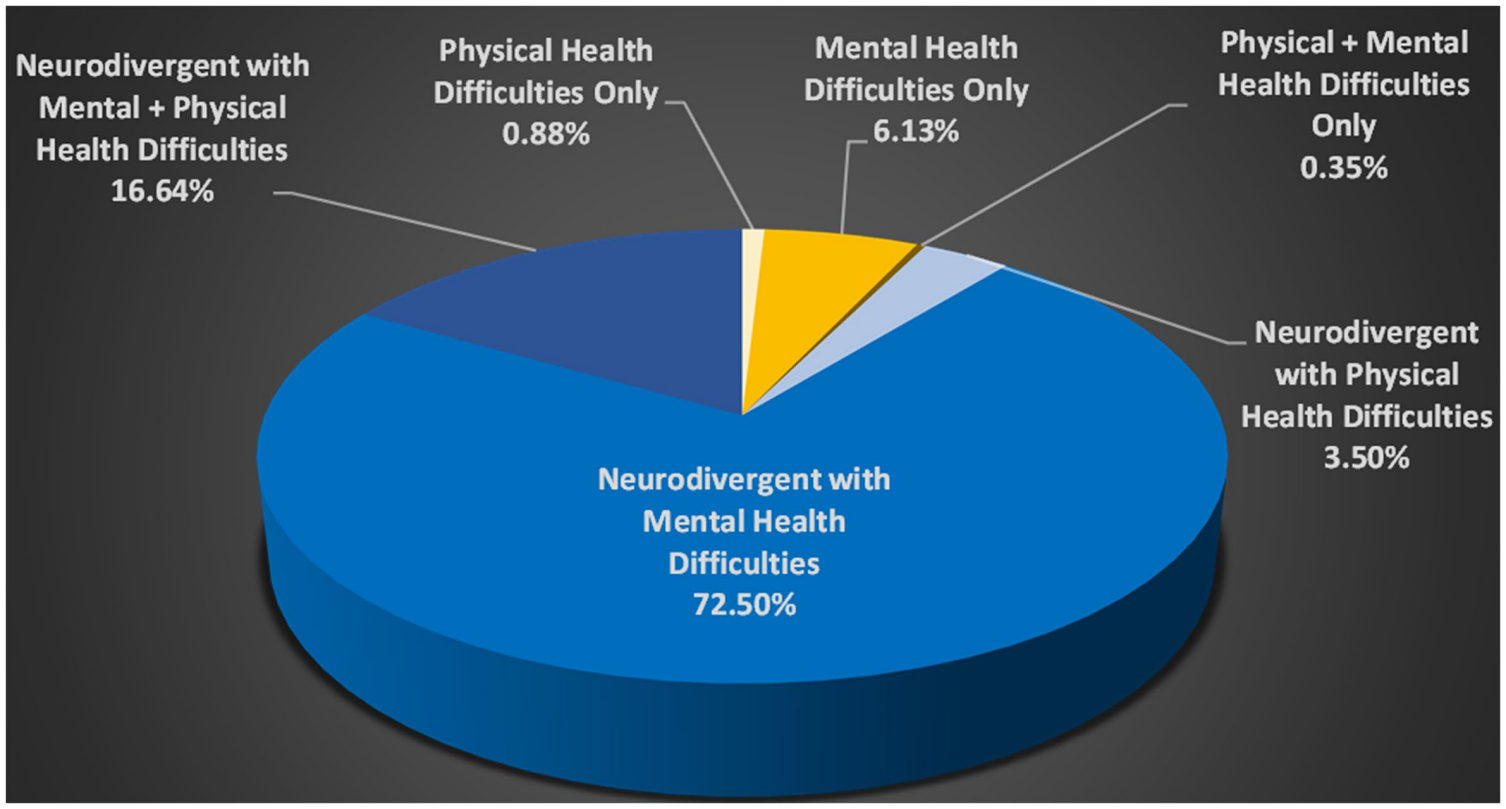
Health Difficulties in the Current School Distress Group (as listed by parents in an optional free text box) x Neurodivergence. The working definition of neurodivergence used in this research recognizes all conditions listed in Table 2 (including sensory processing differences) as part of the spectrum of neurodivergence. Health Difficulties include the conditions described in Table 4.

These responses were further subdivided into three categories: CYP whose parents listed MH difficulties only, whose parents listed physical health difficulties only, and whose parents listed both mental and physical health difficulties. Rates of CYP whose parents reported physical health conditions in isolation were relatively low in all groups (see Supplementary Table S3; Figure 4). However, having either MH difficulties in isolation, or in combination with physical health difficulties, was strikingly more common in both School Distress groups than in both the No School Distress and Lifelong EHE groups. No formal statistical analyzes were conducted here as more precise data (including anxiety data gathered using a clinical scale) is described below. Co-occurrence between neurodivergent conditions and health difficulties is also discussed further below.

#### 3.4.2. Neurodivergence

Neurodivergent CYP were significantly over-represented among CYP who currently experience, or who have previously experienced, School Distress, with 92.05% of the Current School Distress group rated as neurodivergent by their parents (i.e., “Yes” or “Maybe”), compared to 22.2% of those without School Distress experience. Statistically, Current School Distress CYP were significantly more likely to be neurodivergent than Past School Distress CYP, and Past School Distress CYP were significantly more likely to be neurodivergent than No School Distress CYP (Current SD > Past SD > No SD). Notably, the OR for a CYP to experience School Distress if they are neurodivergent was 32.57 (95% CI 20.903, 50.762). Restricting the criteria of ND to just the CYP whose parents responded “yes” increased this OR to 42.25 [95% CI (24.53, 72.78)].

Lifelong EHE CYP were equally likely to be described as neurodivergent by their parents as CYP in both School Distress groups [(Current SD=Past SD = Lifelong EHE) > No SD, *p* < 0.008 (Bonferroni adjusted alpha level)]. The OR for a CYP in the Lifelong EHE group being neurodivergent (considering “yes” and “maybe” responses) relative to a CYP in the No School Distress group was 25.8 (95% CI 7.26, 91.46), suggesting the Lifelong EHE CYP had neurodevelopmental profiles comparable to the CYP in the two School Distress groups.

Co-occurrence between neurodivergent conditions was high, with many CYP having multiple neurodivergent conditions [overall mean = 3.14 (StDev = 2.62); Current SD = 3.70 (StDev = 2.51); Past SD = 3.0 (StDev = 2.54), No SD = 0.72 (StDev = 1.73); Lifelong EHE = 2.52 (StDev = 2.0)]. Number of neurodivergent conditions per CYP differed significantly between the four groups [H(2)=218.123, *p* < 0.001], with No School Distress CYP having significantly fewer neurodivergent conditions than all three other groups (all *p*-values<0.001). No significant differences were found between the Lifelong EHE group and either School Distress group.

Co-occurrence between neurodivergent conditions and health difficulties, particularly MH difficulties, was also high. Notably, 89.14% of CYP in the Current School Distress group whose parents listed one or more health difficulty, were also neurodivergent. Having a physical health condition in isolation accounted for only 0.88% of cases of Current School Distress CYP, while having a mental health condition in isolation accounted for just 6.13% of cases (see Figure 4).

#### 3.4.3. Autism

Autism was the most prevalent neurodivergent condition among CYP with School Distress experience (Current SD: 83.4%; Past SD: 66.2%; see Table 2). These prevalence rates include all individuals who were either diagnosed or suspected to be autistic, meaning prevalence rates were lower when analysis included only CYP with a confirmed autism diagnosis (Current SD: 46.9%; Past SD: 42.1%).

Statistically, CYP in both School Distress groups were more likely to be autistic than No School Distress CYP (Current SD > Past SD > No SD; see Table 2 for further details). Notably, the OR of an autistic CYP (suspected or diagnosed) experiencing School Distress (Current or Past) was 37.69 [95% CI (23.22, 61.18)], relative to non-autistic CYP. This increased to 46.61 [95% CI (24.67, 88.07)] when analysis included only autistic CYP with confirmed diagnoses.

Lifelong EHE CYP were also significantly more likely to be autistic than No School Distress CYP (Lifelong EHE > No SD). The odds of a Lifelong EHE CYP having a confirmed autism diagnosis was significantly greater than for No School Distress CYP [OR = 6.44 (95% CI 0.98, 42.46)]. This increased further when non-diagnosed autistic CYP were included [OR = 20.11 (95% CI 5.33, 75.85)].

#### 3.4.4. Sensory processing difficulties

**Figure 5:**
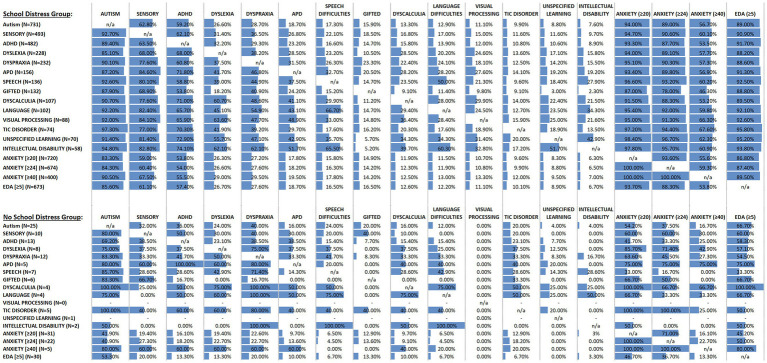
Represents the neurodivergent, anxiety, and extreme demand avoidant (EDA) profiles of the CYP with School Distress (top panel) and without School Distress (bottom panel), and the co-occurrence of these presentations within each group. More specifically, for the column labeled AUTISM, the upper panel indicates that there were 731 autistic CYP in the School Distress Group, while the lower panel indicates that there were only 25 autistic CYP in the No School Distress Group. Next, reading down the column, it is evident that of the 731 autistic CYP in the School Distress Group, 92.7% also had co-occurring sensory processing difficulties, 89.4% also had co-occurring ADHD, 85.1% also had co-occurring dyslexia, etc. relative to the 25 autistic CYP in the No School Distress Group, in which 80% had co-occurring sensory processing difficulties, 69.2% had co-occurring ADHD, 75% had co-occurring dyslexia, etc., Moving across to the SENSORY column, the table shows that 493 CYP in the School Distress group had sensory processing difficulties (upper panel), relative to only 10 CYP in the No School Distress group (lower panel). Moreover, and again reading down this column, the table shows that of the 493 CYP with sensory processing difficulties plus School Distress (upper panel), 62.8% were also autistic, 63.5% also had ADHD, 68% also had dyslexia, etc. This can be contrasted with the 10 CYP with sensory processing difficulties in the No School Distress group (lower panel), whereby only 32% of these CYP were also autistic, 38.5% also had ADHD, 37.5% also had dyslexia, etc.

**Figure 6:**
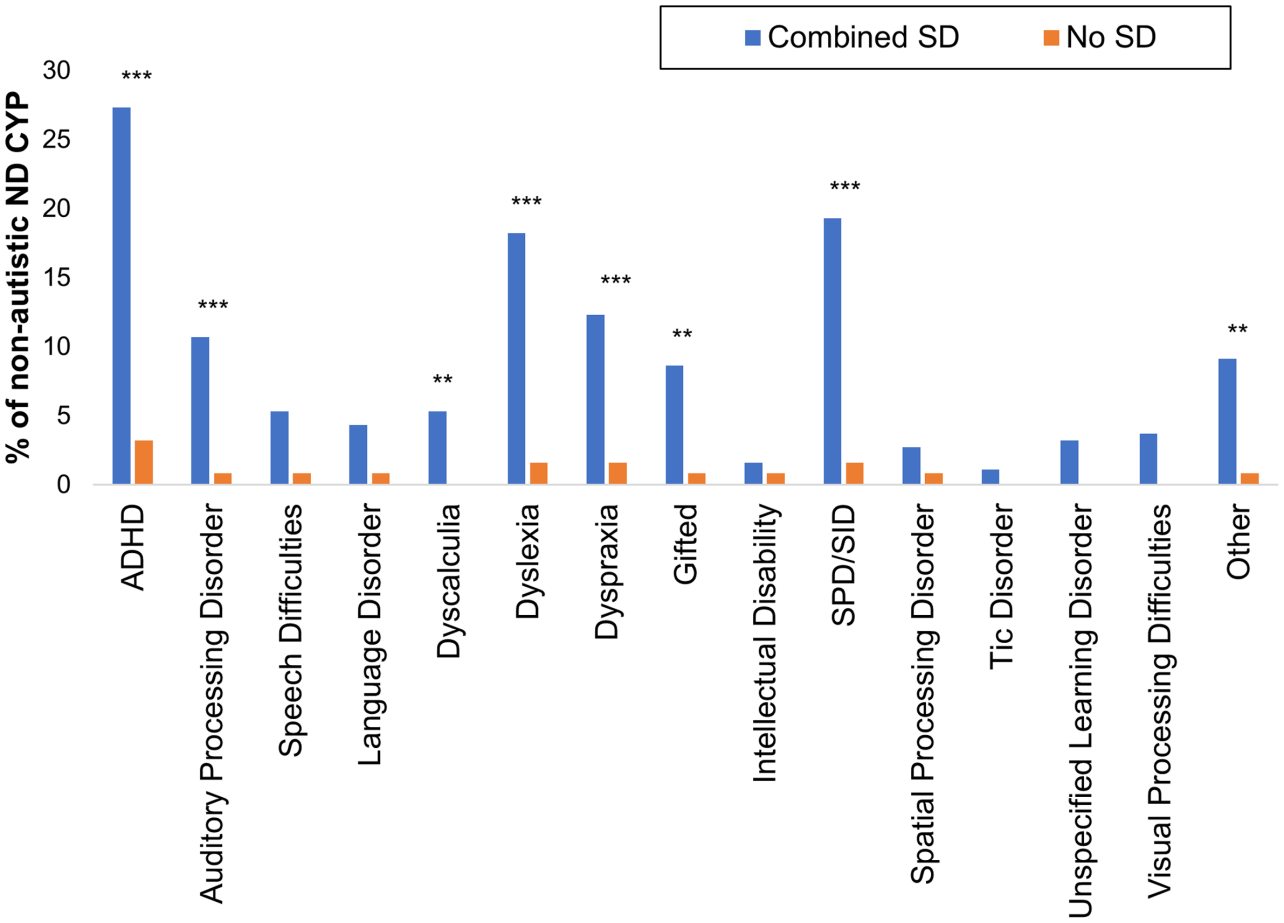
Neurodivergent conditions identified among non-autistic CYP with and without School Distress experience. ‘Combined SD’ includes children in the Current and Past School Distress groups. *p < 0.05; **p < 0.01; ***p < 0.001.

The second most prevalent neurodivergent condition among CYP with School Distress experience was Sensory Processing Disorder/Sensory Integration Disorder (SPD/SID), and again, prevalence differed significantly between groups (see Table 2). Visual inspection of Figure 5 (column 2) shows the markedly increased prevalence of SPD/SID in CYP with School Distress (top panel) relative to those without School Distress (bottom panel), across the breadth of neurodivergencies and differing levels of anxiety and demand avoidance (with only a few exceptions). CYP within the Lifelong EHE group were also significantly more likely (*n* = 13/25) than CYP without School Distress (*n* = 5/50) to have SPD/SID [*χ*^2^(1) = 14.501, *p* < 0.001; analysis conducted using the Lifelong EHE CYP and their age-matched No School Distress group]. Moreover, restricting the dataset to only non-autistic CYP, SPD/SID remained significantly more prevalent in CYP with School Distress relative to those without School Distress (*χ*^2^ = 21.627, *p* < 0.001; see Figure 6).

In cases where SPD/SID was indicated, difficulties were reported in an average of 4.8 sensory systems (StDev = 2.1) and having difficulties in just one sensory system was rare, accounting for 3.7% of reported cases. When split by group, the mean number of systems impacted was 4.79 for Current School Distress CYP (StDev = 2.08), 4.96 for Past School Distress CYP (StDev = 2.09), 4.1 for No School Distress CYP (StDev = 2.6) and 4.62 for Lifelong EHE CYP (StDev = 2.53). The tactile system, followed closely by the auditory system (both>80%), were the systems identified most frequently as being impacted (see Supplementary Table S4, upper panel).

Across all CYP (including those for whom SPD/SID was not reported), difficulties were reported in an average of 2.28 (StDev = 2.8) sensory systems [Current SD = 2.72 (StDev = 2.8); Past SD = 2.19 (StDev = 2.8); No SD = 0.27 (StDev = 1.2); Lifelong EHE CYP = 2.61 (StDev = 3)]. A Kruskal-Wallis test indicated the number of systems impacted differed between groups, H(3)=111.340, *p* < 0.001, with pairwise comparisons revealing: Current School Distress (Mdn = 2) > No School Distress (Mdn = 0), *p* < 0.001; Past School Distress (Mdn = 0) > No SD, *p* < 0.001; and Current SD > Past SD, *p* = 0.046.

For CYP experiencing School Distress, the number of sensory systems impacted (range: 0–8) correlated significantly with School Distress duration (rs = 0.153, *p* < 0.001), age of onset of School Distress (rs = −0.214, *p* < 0.001), and school attendance in the previous 4 weeks (rs = −0.141, *p* = 0.002), 2021/22 academic year (rs = −0.199, *p* < 0.001), and 2020/21 academic year (rs = −0.137, *p* = 0.003). Number of sensory systems impacted also correlated with anxiety (rs = 0.422, p < 0.001), EDA (rs = 0.403, *p* < 0.001), and the degree of emotional distress associated with school attendance (rs = −0.319, *p* < 0.001).

When the dataset was split into autistic and non-autistic but otherwise neurodivergent groups, significant differences in frequency of SPD/SID were observed [*χ*^2^(1,880) = 25.648, *p* < 0.001]; with 61.6% of the autistic CYP experiencing SPD/SID relative to only 36.8% of the non-autistic neurodivergent CYP. Moreover, when SPD/SID was indicated, the mean number of sensory systems impacted for autistic CYP (mean = 4.89, StDev = 2.08, Mdn = 5) was significantly greater than for non-autistic neurodivergent CYP (mean = 3.81, StDev = 2.23, Mdn = 0, U = 56871.5, *p* < 0.001). Note: the neurotypical group was not included in the former analyzes as the working definition of neurodivergence used in this research recognizes sensory processing differences as part of the spectrum of neurodivergence.

Finally, we explored co-occurring conditions amongst autistic CYP with and without School Distress. We found a significantly higher frequency of SPD/SID in the autistic CYP with School Distress experience [Current and Past School Distress combined] than in the autistic CYP with no School Distress experience (*χ*^2^(1)=9.692, *p*= 0.002).

#### 3.4.5. Other neurodivergent conditions

The third most prevalent neurodivergent condition among individuals with School Distress experience was ADHD, followed by dyslexia, dyspraxia, Auditory Processing Disorder (APD), speech difficulties, and giftedness, while intellectual disability and spatial processing disorder were the least prevalent (see Table 2). Both SPD/SID (see above) and Tic disorders were reported more frequently in autistic CYP (10.2%) than in non-autistic ND CYP (1.8%; *χ*^2^(1,880) = 8.53, *p* < 0.001), and there were no instances where a neurodivergent condition was more prevalent in the non-autistic ND group relative to the autistic ND group (see Table 5).

**Table 5:** Neurodivergent conditions in the autistic CYP and non-autistic neurodivergent CYP.

	Autistic	Non-Autistic ND					
	(*n* = 769)	(*n* = 114)	*χ* ^2^	df	*n*	Sig	No SD
Autism	**100% (*n* = 769)**	**0%**	-	-	-	-	3.25% (*n* = 25)
Sensory	**61.6% (*n* = 474)**	**36.8% (*n* = 42)**	**25.648**	**1**	**880**	**< 0.001**	1.94% (*n* = 10)
ADHD	58.5% (*n* = 448)	51.8% (*n* = 59)	1.841	1	880	NS	2.56% (*n* = 13)
Dyslexia	26.2% (*n* = 201)	32.5% (*n* = 37)	1.943	1	880	NS	3.36% (*n* = 8)
Dyspraxia	28.9% (*n* = 221)	21.9% (*n* = 25)	2.360	1	880	NS	4.87% (*n* = 12)
APD	18.5% (*n* = 142)	19.3% (*n* = 22)	0.038	1	880	NS	3.05% (*n* = 5)
Speech difficulties	17.6% (*n* = 135)	10.5% (*n* = 12)	3.593	1	880	NS	4.75% (*n* = 7)
Gifted	16.2% (*n* = 124)	17.5% (*n* = 20)	0.133	1	880	NS	4.16% (*n* = 6)
Dyscalculia	13.2% (*n* = 101)	8.8% (*n* = 10)	1.754	1	880	NS	3.6% (*n* = 4)
Language disorder	12.9% (*n* = 99)	7.9% (*n* = 9)	2.331	1	880	NS	3.7% (*n* = 4)
Visual processing difficulties	10.6% (*n* = 81)	6.1% (*n* = 7)	2.168	1	880	NS	0%
Tic disorder	**10.2% (*n* = 78)**	**1.8% (*n* = 2)**	**8.530**	**1**	**880**	**< 0.01**	6.25% (*n* = 5)
Unspecified learning disorder	8.5% (n = 65)	6.1% (*n* = 7)	0.727	1	880	NS	1.39% (n = 1)
Intellectual disability	7.4% (n = 57)	3.5% (*n* = 4)	2.379	1	880	NS	3.22% (*n* = 2)
Other	12% (n = 92)	16.7% (*n* = 19)	1.952	1	880	NS	2.6% (*n* = 3)

For the autistic CYP, these are likely classified as co-occurring conditions. For the non-autistic CYP, these may be single diagnoses or co-occurring conditions. Chi-Square analyzes explored between-group differences in prevalence of individual neurodivergent conditions between the autistic and non-autistic neurodivergent CYP (with the bold text highlighting significant differences). The ‘No SD’ column on the right is for illustrative purposes only and represents the percentage of neurodivergent CYP (out of the total number of neurodivergent CYP in the sample) that did not experience School Distress.

Finally, considering the prevalence of other neurodivergent conditions among non-autistic CYP with and without School Distress, we found a significantly higher frequency of ADHD [*χ*^2^(1) = 29.617, *p* < 0.001], APD [*χ*^2^(1) = 11.579, *p* = 0.001], dyscalculia (*p* = 0.007, Fisher’s Exact test), dyslexia [*χ*^2^(1) = 19.998, *p* < 0.001], dyspraxia [*χ*^2^(1) = 11.518, *p* = 0.001], giftedness [*χ*^2^(1) = 8.666, *p* = 0.003], SPD/SID [*χ*^2^ = 21.627, *p* < 0.001], and ‘other’ neurodivergent conditions [χ^2^(1) = 9.385, *p* = 0.002] among non-autistic CYP with School Distress relative to non-autistic CYP without School Distress (see Figure 6).

#### 3.4.6. Anxiety

**Figure 7:**
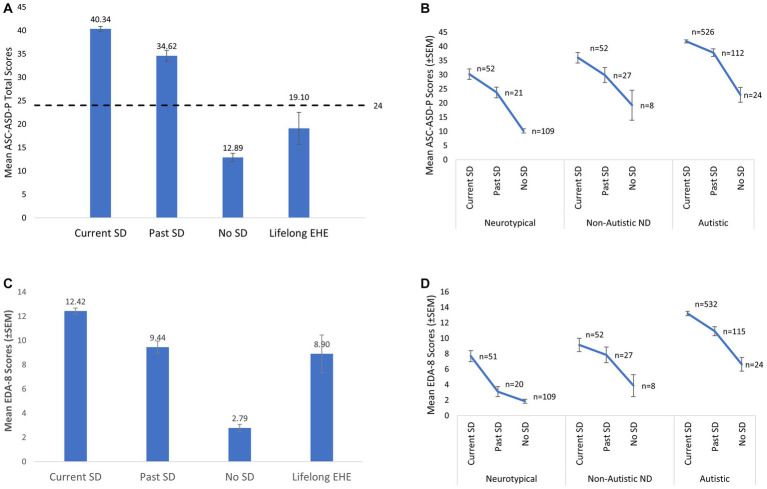
**(A)** Mean total Anxiety scores (ASC-ASD-P) for each of the four groups. The dashed line represents the cut-off score indicative of clinically significant levels of anxiety (i.e., a score of 24). **(B)** Mean total anxiety scores in each group further sub-divided with respect to neurotype. **(C)** Mean Extreme Demand Avoidance scores (EDA-8) for each of the four groups. **(D)** Mean Extreme Demand Avoidance scores (EDA-8) in each group further sub-divided with respect to neurotype. Error bars represent +/− 1 SEM. Non-Autistic neurodivergent = non-autistic, otherwise neurodivergent CYP. The Lifelong EHE group are not represented in B and D due to low numbers (e.g., *n* = 2 in the NT group).

Individual total scores on the ASC-ASD-Parent Version (67) ranged from 0 to 72. Only 7.5% of Current School Distress CYP did not reach the cut-off indicative of significant anxiety symptomatology (i.e., 20), with 92.5% meeting or exceeding this score (see Figure 7A). Moreover, 86.7% of Current School Distress CYP scored above 24 and therefore exceeded the more specific cut-off score for significant anxiety, while 53.8% of Current School Distress CYP scored at least twice the initial cut-off (40+).

There were also significant differences in the frequency of CYP scoring above and below the initial cut-off score of 20 between the Current and No School Distress groups, χ^2^(1, 771) = 353.661, *p* < 0.001. The OR of a CYP experiencing School Distress if a CYP scored 20+wwas 44.015 (95% CI 26.773, 72.362). Moreover, both Current and Past SD groups had significantly higher total anxiety scores than the No School Distress and Lifelong EHE groups, with total anxiety scores highest in the Current SD group [Current SD > Past SD > (No SD = Lifelong SD), see Table 6 for full details]. Additional analyzes using the Lifelong EHE age-matched comparison groups are presented in Supplementary Table S5.

**Table 6:** Between-group comparison of anxiety (ASC-ASD-P) and extreme demand avoidance (EDA-8) total scores.

Measure	Current SD		Past SD		No SD		Lifelong EHE		H(3)	*p*	Significant group differences*
	Median	*IQR*	Median	*IQR*	Median	*IQR*	Median	*IQR*			
ASC-ASD-P Total	41	*19*	33	*20*	10	*12.75*	14.5	*20.75*	296.9	<0.001	Current SD > Past SD > Lifelong EHE and No SD
EDA-8 Total	13	*9*	9	*10*	1	*4*	7.5	*7.75*	242.9	<0.001	Current SD > Past SD; Current SD and Past SD and Lifelong EHE > No SD

*Significance values have been adjusted by the Bonferroni correction for multiple tests. The table displays the medians, Interquartile Range (IQR), and Kruskal-Wallis tests and subsequent pairwise comparisons.

We also explored whether anxiety scores correlated with our markers of School Distress. Higher anxiety correlated significantly with longer School Distress Duration (rs = 0.150, *p* < 0.001), more negative impact of school attendance on MH (rs = −0.545, *p* < 0.001) and lower school attendance in the previous 20 days (rs = −0.41, *p* = 0.002), and the 2021/22 (rs = −0.199, *p* < 0.001) and 2020/21 academic years (rs = −0.137, *p* < 0.001).

However, when CYP (*n* = 951) were re-categorized as a function of broad neurotype [i.e., autistic, non-autistic but otherwise neurodivergent, and neurotypical] as opposed to school attendance difficulties, a significant between-group difference in anxiety was also observed (*H* = 260.70, df = 2, *p* < 0.001). More specifically, anxiety was highest in the autistic group and lowest in the neurotypical group (autistic>non-autistic ND > NT; bonferroni-corrected pairwise comparisons; all *p*-values <0.001). This presence of significantly higher anxiety among autistic CYP relative to neurotypical and non-autistic neurodivergent CYP, regardless of the presence or absence of School Distress, represents a potential confound when interpreting ASC-ASD-P scores as the significantly higher scores in the School Distress groups could have been driven by the significantly different rates of autistic CYP between the groups (see above for more detail), as high anxiety is well documented among such CYP. Hence, we additionally compared anxiety levels between the Current, Past, and No School Distress groups for each neurotype separately. Importantly, significantly higher anxiety in CYP with School Distress was evident in each neurotype [Autistic Group (*n* = 662): H (2)=38.631, *p* < 0.001, Current SD > Past SD > No SD; Non-Autistic ND Group (*n* = 87): H (2)=10.111, *p* = 0.006, Current SD > No SD; Neurotypical Group (n =182): H (2)=85.174, *p* < 0.001, Current SD=Past SD > No SD] (see Figure 7B).

### 3.5. Demand avoidance

The groups also differed significantly with respect to total demand avoidance (EDA-8) scores [H(3)=242.945, *p* < 0.001], with total EDA-8 scores highest in the Current SD group and lowest in the No School Distress group (see Table 6; Figure 7C). Additional analyzes using the Lifelong EHE age-matched comparison groups are presented in Supplementary Table S5.

Higher EDA-8 scores correlated significantly with longer School Distress duration (rs = 0.095, *p* = 0.008), younger age of onset of School Distress (rs = −0.205, *p* < 0.001), more negative impact of school attendance on MH (rs = −0.101, *p* = 0.011), and worse school attendance in the previous 20 days (rs = −0.126, *p* = 0.005) and the 2021/22 academic year (rs = −0.106, *p* = 0.021). Higher EDA-8 scores also correlated significantly with number of sensory systems impacted (rs = 0.402, *p* < 0.001), and higher anxiety (rs = 0.483, *p* < 0.001).

Re-categorizing the CYP with respect to neurotype instead of school attendance difficulties, a significant between-group difference in demand avoidance was also observed (*H* = 49.62, df = 2, *p* < 0.001), with total EDA-8 scores highest in the autistic group relative to both the non-autistic but otherwise neurodivergent group and the neurotypical group [all *p*-values <0.001]. Again, this presence of significantly higher demand avoidance among autistic CYP relative to neurotypical and non-autistic neurodivergent CYP, regardless of the presence or absence of School Distress, represents a potential confound when interpreting our findings as the differences could be driven by the unequal proportion of autistic CYP across the School Distress and no School Distress groups. Hence, we additionally compared EDA-8 scores between the Current, Past, and No School Distress groups for each neurotype separately.

Importantly, significant between-group differences persisted in both the Neurotypical and the Autistic groups, whereby autistic CYP in the School Distress groups had significantly higher EDA-8 scores than autistic CYP in the No School Distress group [H(2)=34.317, p < 0.001, Current SD > Past SD > No SD], and neurotypical CYP in the Current School Distress group had significantly higher EDA-8 scores than neurotypical CYP in the No School Distress group [H(2)=59.009, *p* < 0.001, Current SD > (Past SD=No SD)] (Figure 7D).

## 4. Discussion

This study identified several prevalent characteristics among CYP affected by school attendance problems. In most cases, school attendance problems were underpinned by significant emotional distress associated with school attendance, and parental accounts regarding their child’s difficulties were often harrowing. This led us to devise the term ‘School Distress’ to replace pre-existing terms such as “School Refusal,” as this reinforces that for many CYP, the defining feature of their experience is not a “refusal” to attend school, but rather the severe emotional distress experienced when attempting to do so (see Figure 1). Additionally, definitions of “School Refusal” often require CYP to be absent from school for a period of time [e.g., (71, 72) which specify an absence rate of at least 10–50% in the prior month], thus failing to adequately capture the experiences of many of the CYP described above who experienced School Distress but who continued to attend school despite the emotional distress experienced. Given their unaffected attendance rates, and the prior absence of an adequate typology, these CYP’s distress may fall under the radar of educational professionals, particularly in the emergent stages. Hence, we argue the ‘School Refusal’ label, which captures nothing of the emotional distress suffered by CYP and is deeply unpopular with those with lived-experience of SD (13), should no longer be used. Instead, we propose these difficulties are best described as ‘School Distress’, which is not only person-orientated, but also specifically encompasses CYP who manage to attend school despite their distress.

### 4.1. Children and young people characteristics

The CYP with School Distress experience were young, with onset of their School Distress commonly occurring within their formative years, and their difficulties were enduring. As hypothesized, School Distress first occurred significantly earlier and was more enduring in autistic CYP than in their non-autistic peers, indicating greater School Distress severity. This replicates and extends previous findings showing that school attendance problems occur significantly earlier in autistic CYP (33), and aligns with the findings of Munkhaugen et al. (34), whose teacher reports indicated greater severity of attendance problems in autistic pupils.

The majority of CYP experiencing School Distress either currently or previously attended a mainstream provision. Thus, while not restricted to mainstream provisions, it appears School Distress is common in CYP whose educational journey originated in a mainstream setting, posing the question of whether mainstream settings are suitable for all CYP, and if not, which provisions may be more appropriate.

Consistent with the literature, we did not find compelling evidence of differential rates of School Distress among male and female CYP. Notably, 3.3% of parents identified their child as non-binary or transgender, and 1% selected ‘self-describe’, with these options being more frequently selected by parents of CYP with School Distress experience. Future studies should explore this further to ensure transgender and gender-diverse CYP are being appropriately supported in schools.

Notably, CYP with School Distress were significantly more likely to be neurodivergent than CYP without School Distress, confirming our predictions. This is comparable with Epstein et al. (73) who, in a smaller sample, revealed that about 90% of CYP missing school had Special Educational Needs/Disability (SEND) or a health problem. Similarly, 75% of Amundsen et al.’s (35) participants experiencing school attendance problems were neurodivergent. Co-occurrence of neurodivergencies was high among CYP with School Distress experience, and a large proportion of the CYP experiencing School Distress were neurodivergent and experienced MH difficulties.

The high rate of MH difficulties in our sample is consistent with previous findings showing high levels of depression, anxiety, and stress in CYP experiencing school-related emotional distress [e.g., (45–47)]. They are also consistent with a growing mental health crisis that is reportedly affecting CYP both in the United Kingdom (74, 75) and globally (76). This includes increases in depressive episodes, serious psychological distress and suicide-related outcomes (77, 78), and an almost doubling of school loneliness between 2012 and 2018 (79). However, in the current study, neurotypical CYP experiencing School Distress alongside a MH condition accounted for just 6.13% of cases. Thus, exploring MH difficulties alone [e.g., (46, 47)] is likely to obscure the wider functional profiles of CYP experiencing School Distress, with our data suggesting such CYP predominantly have multiple neurodivergent conditions alongside MH difficulties. Future research should explore the role of neurodivergence in the emergence and maintenance of School Distress further, while simultaneously exploring other factors which could be driving recent secular changes in child and adolescent MH such as increased exposure to social media (80, 81), increased testing in schools (82), and/or a societal shift to knowledge-based economies that increasingly links life chances with educational performance (83). How these latter factors intersect with both School Distress and neurodivergence are also likely relevant.

With respect to neurodivergence in the current study, autism was the most prevalent neurodivergent condition, with significantly higher rates found among CYP with School Distress experience than without, aligning with previous research. Autism prevalence was higher than previous reports [e.g., (33–35)], with Epstein et al. (73) finding just 40% of the CYP missing school in their sample to be autistic. However, previous research has typically only measured diagnosed cases of autism, whereas we included CYP whose autism is diagnosed or indicated. Thus, when we restricted our analysis to include only CYP with a confirmed diagnosis, our prevalence rates were more comparable with those in previous research. Unfortunately, such a method misses the large number of CYP who are currently on the assessment pathway, which typically only occurs after considerable evidence of autism has been compiled across settings [173 CYP in the Current SD group (23.4%) and 20 CYP in the Past SD group (9.6%)]. This also excludes CYP who have had their referral rejected before assessment, which typically occurs due to services requesting more evidence prior to acceptance, and CYP for whom a referral has not yet been made. As previous research has found no significant differences in autism characteristics between adults with a confirmed diagnosis and those who self-identify as autistic or are awaiting diagnosis (84, 85), coupled with the very considerable waiting times for an autism assessment in the United Kingdom (86), we argue broader inclusion criteria are likely to provide a more accurate estimation of the prevalence of autism among CYP with School Distress.

Sensory processing difficulties (i.e., SPD/SID) were the second most prevalent neurodivergence among our School Distress samples and were significantly more prevalent in the School Distress groups relative to No School Distress CYP. Additionally, sensory processing difficulties were significantly more prevalent in autistic CYP with School Distress than in autistic CYP in the No School Distress group. Finally, although autistic CYP were significantly more likely to have SPD/SID than non-autistic neurodivergent CYP, over a third of non-autistic neurodivergent CYP were still classified as having SPD/SID. Hence, CYP with SPD/SID, and in particular autistic CYP with SPD/SID, may have a particular vulnerability to School Distress. Given that co-occurring sensory processing difficulties appear to increase risk of School Distress in autistic children, this may offer one potential explanation as to why only some autistic children experience School Distress (34).

Difficulties within a single sensory system were rare, with CYP with SPD/SID having difficulties across an average of 4.8 sensory systems. Critically, School Distress CYP had difficulties in significantly more sensory systems than CYP with no School Distress and the number of sensory systems impacted correlated significantly with anxiety and all proxy markers of School Distress. This supports the hypothesis that more pervasive sensory difficulties are associated with more severe School Distress and extends past research that identified the overwhelming sensory demands of the school environment as reasons why CYP with sensory processing difficulties can find school distressing (36, 39, 41, 87, 88). Further reinforcing the potential role played by sensory processing difficulties in School Distress was the observation that just 1.9% of the CYP reported to experience sensory processing difficulties fell into the No School Distress group. Hence, having no School Distress was extremely rare among the CYP identified by their parent/carer as having SPD/SID.

Interestingly, difficulties were noted in the tactile and auditory systems in 4/5 CYP with SPD/SID. Tactile hypersensitivity and auditory filtering have previously been linked to cognitive inattention and academic under-performance in autistic CYP in mainstream classrooms (89), potentially providing insight into why individuals with SPD/SID are at increased risk of School Distress. Relevant also are Howe and Stagg’s findings (90) that autistic pupils attending mainstream school perceived auditory differences to be most disruptive to their learning, followed by touch, smell, and vision. Furthermore, difficulties in the olfactory system were noted in 2/3 CYP with SPD/SID in our study, resonating with observations that “PE changing room” and “incidental smells such as perfume and cleaning products” are particularly challenging sensory experiences for autistic pupils in school (36) (p. 7). Such olfactory processing difficulties, alongside differences in the gustation system (indicated in half of the CYP with SPD/SID reported here), may also explain why many autistic CYP find school halls/canteens particularly distressing. The sensory difficulties identified in this study align with the findings of Jones et al. (36) who explored the impact of SPD on autistic pupils’ learning and school life, with parental comments including: “They try to protect themselves by covering their ears, closing their eyes, pulling their t-shirts over their noises to block out smells.” In order to fully elucidate the role played by SPD in School Distress, future studies should seek to further assess the severity of these difficulties, the specific systems in which they occur, and explore these parameters with respect to anxiety, demand avoidance, autism, and neurodivergent more broadly.

ADHD, APD, dyscalculia, dyslexia, dyspraxia, giftedness, SPD/SID, and ‘other’ neurodivergent conditions were significantly more prevalent in non-autistic School Distress CYP relative to non-autistic CYP with No School Distress, indicating that these neurodevelopmental differences may increase risk of School Distress in the absence of autism. Given the high co-occurrence of neurodivergent conditions in this study, coupled with autism being so prominent among School Distress CYP, care is however needed when interpreting the individual impact of each neurodivergent condition on School Distress. Interestingly, the high co-occurrence of neurodivergent conditions in our School Distress sample may be a key finding in itself; whereby it may be the complexity of managing multiple neurodivergent conditions within an environment optimized for the neurotypical learner that overwhelms these CYP and renders the school environment so difficult and detrimental to their wellbeing. Support and planning will likely therefore need to be multidimensional and bespoke to the specific needs of individual CYP experiencing School Distress. Future studies may seek to fully explore the prevalence of School Distress in CYP with neurodivergent conditions such as ADHD, dyslexia, and dyspraxia, all of which were present at relatively high rates in our School Distress groups.

The disproportionate rates of neurodivergence found among School Distress samples are extremely concerning and indicate that appropriate support for neurodivergent CYP needs to be improved. Relatedly, care needs to be taken to ensure CYP with School Distress have access to timely assessments of underlying neurodivergent conditions, with autism, SPD/SID, ADHD, dyslexia, dyspraxia, and EDA (among others) all important to consider. It is also critical that educators and policy makers consider whether mainstream schools are truly inclusive environments for CYP with SEND more broadly. Indeed, D’Alessio (91) highlighted that SEND CYP are frequently segregated from peers, resulting in feelings of exclusion within mainstream environments, while Webster et al. (92) described the experience of high-needs SEND CYP as often equating to “*marginalization masquerading as mainstream”* (p. 77).

Of additional concern is the finding that over 90% of Current School Distress CYP met or exceeded the cut-off indicative of significant anxiety symptomatology on the ASC-ASD-P. Moreover, CYP in both School Distress groups had significantly higher anxiety than CYP who have never experienced School Distress, regardless of neurotype. When grouped with respect to neurotype and School Distress experience, the autistic Current School Distress group had particularly high anxiety scores (mean = 41.52, which is over twice the cut-off score for significant anxiety). Such scores are markedly higher than previously published scores using the ASC-ASD-P in autistic CYP (93). Such elevated scores are of concern, not least because higher anxiety severity is associated with a lower quality of life in both autistic and non-autistic children with anxiety disorders (94), and in autistic CYP more generally (93, 95). Hence, supporting individual CYP experiencing such levels of anxiety should be a priority for educational and health-care professionals. Overall, while these findings replicate those of Gonzalvez et al. (47) who also found significantly higher anxiety levels among school “refusing” CYP compared to CYP without school attendance problems, it also extends previous research by using a larger sample size and a broader typology, and considers CYPs’ neurodevelopmental profiles. Moreover, it builds on previous research by using a clinical scale devised using evidence of the anxiety phenomenology in autistic CYP specifically, including items relating to sensory anxiety, intolerance of uncertainty, and phobias (67). This is important as anxiety symptoms differ in the context of autism (96), and autistic CYP appear to be at considerably greater risk of School Distress. Of note, mean anxiety scores in autistic CYP in the No School Distress and Lifelong EHE groups also exceeded the cut off for significant anxiety, demonstrating the more general heightened anxiety in autistic CYP, however these scores were markedly lower than in the School Distress groups. Finally, when exploring how anxiety related to our proxy markers of School Distress, we found higher anxiety significantly correlated with more extensive School Distress, greater level of emotional distress due to school attendance, and lower school attendance in the previous 20 days, and in the 20/21 and 21/22 academic years. Such high anxiety among CYP with School Distress may be a cause or consequence of School Distress, or both.

As anticipated, School Distress CYP scored significantly higher on the EDA-8 (69) than No School Distress CYP. Hence, consistent with parental accounts, CYP with School Distress appear to display significantly more EDA behaviors than CYP who attend school without difficulties. Additionally, scores on the EDA-8 correlated significantly with all proxy markers of School Distress severity and higher anxiety, reinforcing the link between EDA and anxiety, and supporting previous anecdotal and research links between Demand Avoidance and school attendance problems (59, 60). Interestingly, autistic CYP had, on average, higher EDA scores compared to both the NT and non-autistic neurodivergent groups, confirming previous accounts linking high levels of demand avoidance to the autistic experience.

In addition to the above correlations, scores on the EDA-8 also correlated with the number of sensory systems in which CYP experience difficulties. These relationships warrant further consideration to understand why and how demand avoidant behaviors become so pervasive in autistic CYP (and indeed in some otherwise neurodivergent and neurotypical CYP), and how they relate to the emergence and maintenance of School Distress. This could help address the current dearth of understanding of how best to support CYP with demand avoidant profiles in traditional education settings, particularly in mainstream school environments that so often fail to provide inclusive sensory environments for CYP who experience considerable sensory distress (40).

Strikingly, almost one-third of parents in the Current School Distress group reported that their child received no support at school. Moreover, for many of those CYP who were on the school’s SEN register (or equivalent) or had an EHCP, this did not translate into ring-fenced SEN support at school, as indicated by parental comments, with many parents also referring to a lack of support from their child’s school when they attempted to seek additional support, and schools blocking their attempts to secure an EHCP for their child. The likely reason for this is well documented elsewhere, i.e., the issues of school budgets and the fact that English schools must pay the first £6,000 of meeting an EHCP from their own budgets annually (97, 98).

### 4.2. Birth order and sibling school attendance problems

Within this study, we replicated a previous observation in the literature which noted that a high rate of CYP who experience school attendance problems are the youngest child in their family (99). This was particularly evident in our Current School Distress group, where almost 40% of CYP were the youngest in their families. Given that we also found that youngest CYP with School Distress often had an older sibling(s) who had also experienced attendance problems (Supplementary Figure S1), the high rates of youngest children experiencing School Distress observed here may well be due to multiplier effects, whereby genetic (e.g., ND) and environmental factors (e.g., experience of a previous sibling’s School Distress, specific SEND provision in the local schools) interact and compound the risk of subsequent children in the family experiencing School Distress.

### 4.3. Lifelong EHE CYP and their families

CYP whose parents had decided school-based education was not appropriate for them at an early point in life (Lifelong EHE) showed similar neurodevelopmental profiles to School Distress CYP, with comparable rates of neurodivergence. Lifelong EHE CYP were also significantly more likely to have both sensory processing difficulties and elevated demand avoidant scores relative to No School Distress CYP. Importantly, however, there were no significant differences with respect to anxiety scores between the Lifelong EHE CYP and the No School Distress CYP, and both of these groups had significantly lower anxiety scores than the Past and Current School Distress groups, indicating that despite comparable neurodivergence and demand avoidant profiles in the Lifelong EHE CYP, these young people were not experiencing the same levels of anxiety as the CYP who experienced School Distress (be that currently or historically). Relatedly, while 92.2% of Current School Distress and 84.5% of Past School Distress CYP met the clinical cut-off indicative of significant anxiety on the ASC-ASD-P, only 40% of Lifelong EHE CYP met this score. Hence, Lifelong EHE CYP, and particularly neurodivergent Lifelong EHE CYP, are an important comparison group here, and suggest that neurodivergent profile alone is not sufficient to account for these markedly different anxiety profiles. Future research should attempt to explore the emergence of anxiety in both school and home educated ND/demand avoidant CYP longitudinally, to more fully understand the drivers of this anxiety.

Additionally, some of the parents of Lifelong EHE CYP explicitly stated that their child’s neurodevelopmental profile was a key determinant in their decision not to enrol their child in a school setting. These parents noted recognizing early in their child’s life that, given their child’s neurodivergent profile, they would likely face difficulties accessing school-based education (see Supplementary Table S2, Q5). Moreover, some reported that they considered EHE a better fit to their child’s needs as it affords them flexibility to readily adapt their approach to meet the child’s individual learning needs (see Supplementary Table S2, Q6), or to provide the levels of individual support required (see Supplementary Table S2, Q7). This flexibility attunes with the advice of specialist PDA educators (62). However, home education is simply not a feasible educational option for many families, not least due to the considerable financial implications. Moreover, on a societal level, is it acceptable for education outside of the family to be inaccessible to some CYP, simply as a consequence of their neurodivergent profile?

### 4.4. Limitations

One key limitation is the lack of diversity among our sample, with an over-representation of white CYP, meaning our findings may not accurately represent the profiles of all CYP experiencing School Distress. Our Current and No School Distress groups were also living in less deprived areas than would be expected by chance alone. Thus, future research should aim to collect more diverse samples to gain a more comprehensive understanding of the experience of School Distress across all CYP, and to therefore guide more individualized support. Such research may identify different drivers of school attendance problems.

Moreover, this study was limited to the United Kingdom, further reducing the generalisability of our findings. As education systems vary internationally, the School Distress experience and the characteristics of individuals experiencing School Distress may differ between countries, providing an additional avenue for future research.

Additionally, while this study was advertised widely on social media (with a considerable number of post impressions, reaches and engagements), the sites where it was shared may have influenced who participated. For example, within our No School Distress group, 16.8% of CYP were autistic, despite the national prevalence rates of autism standing at around 1–2% (100), indicating this group may not be entirely representative of all CYP who do not experience School Distress. The advertisement was however shared on the largest family support page for families of children and young people facing barriers to school attendance (for whatever reason) in the United Kingdom, i.e., Not Fine in School. Future studies should formally record the social media site that each respondent comes from.

Alternative recruitment strategies include sampling directly from schools, e.g., by contacting parents whose children have a greater number of absences. However, whilst such an approach may confer certain advantages, it would miss the CYP who have fallen out of the school system (101), and hence may only capture milder, or emergent, forms of School Distress. As the data presented here indicates that School Distress is more enduring and associated with higher levels of anxiety and demand avoidance in autistic CYP, sampling milder presentations would likely result in autistic CYP being underestimated. Sampling via schools would likely also miss the CYP who, despite experiencing considerable School Distress, manage to maintain attendance at school, as these CYP may fall under the radar of educational professionals. Moreover, school attendance problems may be the first indicator to professionals that a CYP is not managing in their environment, and it may take some time before the underlying driver of this is identified (e.g., autism, ADHD, dyslexia, etc.), resulting in some emergent cases of School Distress initially being mis-attributed solely to MH difficulties. Indeed, a recent meta-analysis indicated that only 72.4% of cases of autism and 56.8% of cases of ADHD have been recognized by age 14; figures that rise to 89.8% by age 18 and 94.8% by age 25 for autism, and 73% by age 18 and 91.8% by age 25 for ADHD (102).

Online recruitment via social media also enabled us to attempt to capture the full age range of CYP attending United Kingdom schools. While this is a likely strength of the study, it needs to be noted that the age range of the specific sample will likely influence the characteristics. Hence these findings may not extrapolate fully to a more specific age range and, in particular, to mid to late adolescents. This is because, firstly, the overall peak age of onset of mental health difficulties does not occur until 14.5 years of age (and the mean age of the CYP in the current study was just 11.6 years of age), and secondly, because the peak age of onset of MH difficulties varies considerably depending on MH subtype [e.g., 5.5 years of age for anxiety/fear-related disorders relative to 20.5 years of age for mood disorders (102)]. In addition, environmental factors that appear to place downward pressure on CYPs’ MH, such as social media use (81), vary depending on age, with current estimates indicating that 33% of 5-7-year-olds relative to 97% of 16-17-year-olds use social media (103). Moreover, there appear to be distinct developmental windows of sensitivity to social media in adolescence, i.e., 11–13 and 19 years old for females and 14–15 and 19 years old for males (80). Hence, both age and sex of the cohort sampled may impact the observed characteristics.

Notably, we used a broad criterion for neurodivergent conditions, including CYP currently awaiting assessment/diagnosis, CYP whose referral was rejected, and CYP who have yet to be referred. The rationale behind this is discussed at length above, however it is possible this led to an overestimation of prevalence rates. A final weakness of this study is the differing sample sizes between participant groups, with the Lifelong EHE group being particularly small, potentially influencing the accuracy of between-group comparisons. Future research should collect more evenly sized participant groups, although this is challenging in rarer groups such as Lifelong EHE CYP. This disparity did, however, arise due to the volume of CYP currently experiencing School Distress in the United Kingdom.

One key drawback of the School Distress literature generally, as opposed to this study specifically, is the lack of a standardized questionnaire to assess School Distress which is suitable for use in autistic individuals. Given the prevalence of autism among CYP experiencing School Distress, development of such a questionnaire which can be used in clinical, education, and research settings is vital. Thus, one next step should be to gather perspectives of autistic CYP and autistic adults, and to work collaboratively with them to develop a standardized questionnaire to assess School Distress severity and/or risk.

This study also had several strengths, including the large sample of CYP currently experiencing School Distress, and the exploration of various aspects of School Distress within this large sample size. This was much greater than in previous research, enabling stronger conclusions to be made.

## 5. Conclusion

The Human Rights Act states “No person shall be denied a right to an education” (104). However, our findings suggest that a large number of CYP are being denied access to education due to the emotional distress they experience at school. The finding that particular groups of individuals with a recognized disability (e.g., autistic CYP) are specifically affected is of grave concern, particularly when one considers the Human Rights Act (104), the Equality Act (105), the Children Act (106), and Articles 23 and 28 of the United Nations Convention on the Rights of a Child (107), which enshrine all CYPs’ right to an education regardless of disability.

Further research involving both autistic and non-autistic CYP who experience School Distress, and their families, is urgently needed to further understand this problem, and develop solutions that ensure all CYP can access education, and that all families have the option to avail of safe educational opportunities for their child outside of the family. Such research should be used to inform future changes to education legislation, as legislation changes which do not consider this evidence-base run the risk of making the current situation even worse for those CYP at risk of distress-based school attendance difficulties.

Wider discussion with respect to the appropriateness of traditional, school-based education for all CYP is needed. Further research, ideally co-produced with autistic and otherwise neurodivergent individuals, is needed to determine best practices in education, and to ensure appropriate understanding of how neurodivergent pupils best learn (58). Relatedly, research into best-pedagogical practice for pupils with SEND, including pupils with complex presentations, is urgently needed, especially within mainstream settings (108). Given the substantial heterogeneity in the neurobiology of autism, this will undoubtedly be complex, and efforts must include consideration of how learning needs will vary with neurosubtype (109, 110) and demand avoidant profile (57). Research exploring educational and life outcomes of Lifelong EHE CYP, and CYP with provisions such as EOTAS, is also urgently required to better understand how CYP can be successfully educated outside of school settings.

## Data availability statement

The raw data supporting the conclusions of this article will be made available by the authors, without undue reservation.

## Ethics statement

The studies involving humans were approved by Faculty of Medical Sciences Research Ethics Committee, part of Newcastle University’s Research Ethics Committee. The studies were conducted in accordance with the local legislation and institutional requirements. The participants provided their written informed consent to participate in this study.

## Author contributions

SM and SC conceptualized and designed the study, organized the database, and performed the initial statistical analysis. SC, HC, and SM performed the final statistical analyzes. SC and SM wrote the initial drafts of the manuscript. All authors contributed to the article and approved the submitted version.

## Footnotes

^1^using https://www.fscbiodiversity.uk/imd/
