# Supplementary material for: School distress and the school attendance crisis: a story dominated by neurodivergence and unmet need
Source: Front Psychiatry. 2023 Sep 22;14:1237052. doi: 10.3389/fpsyt.2023.1237052 (PMC10556686; doi:10.3389/fpsyt.2023.1237052)
Supplement: Supplementary file 1 [file Data_Sheet_1.pdf]

School distress and the school attendance crisis: A story dominated by neurodivergence and unmet need.

Supplementary Material

Additional organisations that shared the study advertisement on their social media sites included:

- SEND National Crisis,
- Sunshine Support,
- South Tyneside SENDIASS,
- the Kayaks Community,
- the North East Autism Family Network,
- the Toby Henderson Trust,
- the Sensory Place,
- Scottish Boarders and Northumberland PDA,
- Ayrshire PDA Awareness,
- Spectrum Life Scotland, and
- Play Therapy Northumberland.

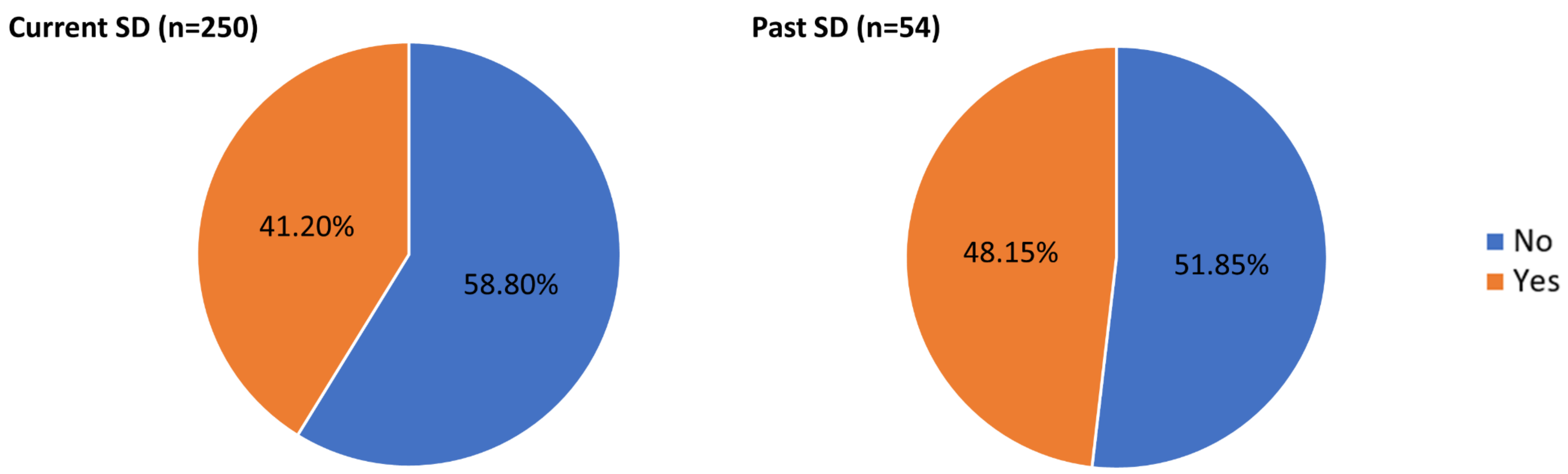

Figure S1. Presence of school attendance problems in any of the siblings of the CYP in the Current and Past School Distress (SD) groups who were the youngest child in their family.

Table S1.

Type of School Attendance Problems. *Reflects the number and percentage of parents who reported that their child's school attendance problems were either fully or partially emotionally based.

Variable	Combined SD	Current SD	Past SD
Type of School Attendance Problems (%)			
Self-corrective	134 (15.55)	105 (15.37)	29 (16.20)
Acute	198 (22.97)	172 (25.18)	26 (14.53)
Chronic	210 (24.36)	183 (26.79)	27 (15.08)
Other	320 (37.12)	223 (32.65)	97 (54.19)
Emotionally Based School Attendance Problems (%)*	813 (94.32)	648 (94.88)	165 (92.18)

Table S2

Additional Quotes Provided by Parents Throughout the Survey in Response to Various Questions. CYP = Children and Young People. SD = School Distress. SEN = Special Educational Needs. EHCP = Education, Health and Care Plan. CSP = Co-ordinated Support Plan. IEP = Individual Education Plan. CAMHS = Child and Adolescent Mental Health Service. EHE = Elective Home Education. ND = Neurodivergent.

Reference	Summary	Example Quotes
Q1	Many comments from parents of CYP with SD experience described a lack of support in place for their child	"no support and attends at less than 10%"<br>"Very limited support from school"<br>"Very little support is given"<br>"We have had TAF [Team Around the Family] meetings but the deputy head was not in support and stopped these"
Q2	Even when parents indicated that their child was on the school's SEN register (or equivalent) or had an EHCP in place, parental comments continued to indicate a lack of support for many CYP	"On SEN register at school but has no actual support"<br>"Is on the SEN register however no further support in school. Nothing in place"<br>"My child received support and was on the SEN register at Junior School but on moving to Secondary this wasn't recognised, accepted or acknowledge...No support whatsoever!"<br>"CSP but no real support"<br>"IEP in school but [needs] not being met"<br>"Has an EHC statement but very little if any support"<br>"EHCP recently received but specified support is unsuitable and not being provided"<br>"Off since Dec. Reassessment refused. Annual review also refused ehcp change... inappropriate "type of school" in section I"<br>"Has ehcp but currently homeschooling due to no suitable setting available and previous setting causing trauma due to excessive restraint and not meeting basic needs"
Q3	Application for and implementation of EHCPs was a particular source of frustration for parents	"I am in the process of applying for this [EHCP] myself as school are unwilling"<br>"Parent application [for EHCP] no support from school"<br>"In mediation. I'd to self-apply [for EHCP] as school delayed and blocked"<br>"Has EHCP but is ignored"<br>"School not following EHCP"<br>"Not that it [EHCP] was followed"<br>"School not named as current school can't meet needs. EHCP done by parental request"<br>"School has put some reasonable adjustments in place after GP letter and a fight"
Q4	Occasional comments reflected a positive situation regarding support for CYP	"My child's school currently provides reasonable adjustments for my daughter's needs while we await her ASC assessment"<br>"Working with the school to establish Sen support/be in the Sen register"<br>"After years of struggling school has finally applied for EHCP"<br>"My child is from Wales, has a statement, now attends out of county independent specialist school in England. Finally."<br>"The EHCP states elective home education with personal budget. Which covers 6 hours tutors and 6 hours 121 forest school."<br>"Some support at school. Under CAMHS"

Q5	Some Lifelong EHE parents recognised early in their child's life that, given their ND, they would likely face difficulties accessing school-based education	"I am certain school would be damaging to my EHE child"
Q6	Some Lifelong EHE parents considered EHE a better fit to their child's needs as it affords them flexibility to readily adapt their approach to meet the child's individual learning needs	"As we EHE, adaptation to individual needs is inbuilt"
Q7	Some Lifelong EHE parents articulated that they considered EHE a better fit to their child's needs as it enables them to provide the high level of support required.	"[CYP is] electively home educated due to needs, so has 1:1 support"

Table S3.

Percentage of Physical and Mental Health Difficulties per Group. Note: Neurodivergencies such as autism, ADHD, dyslexia and sensory processing difficulties were excluded as they were assessed separately. The "None Listed" category includes CYP who do not have any health difficulties AND CYP whose parents opted not to specifically describe these difficulties. SD = School Distress. EHE = Electively Home-Educated.

	Physical Health	Mental Health	Both	None Listed
Current SD	3.73%	67.01%	14.48%	16.27%
Past SD	7.53%	49.46%	6.45%	36.56%
No SD	4.05%	10.14%	1.35%	84.46%
Lifelong EHE	8.33%	25%	0%	66.67%

Table S4.

Sensory Processing Difficulties: Sensory Systems Affected. SPD = Sensory Processing Disorder. SD = School Distress. EHE = Electively Home-Educated. CYP = Children and Young People.

Sensory System	Current SD	Past SD	No SD	Lifelong EHE
CYP with SPD				
Tactile	84.6%	87.1%	80.0%	92.3%
Auditory	82.6%	81.2%	90.0%	84.6%
Interoceptive	67.4%	60.0%	70.0%	61.5%
Olfactory	67.4%	60.0%	70.0%	61.5%
Proprioceptive	52.0%	63.5%	50.0%	69.2%
Gustation	51.7%	55.3%	30.0%	53.8%
Visual	41.3%	40.0%	20.0%	23.1%
Vestibular	38.8%	42.4%	30.0%	30.8%
All CYP				
Tactile	48.1%	38.3%	5.4%	52.2%
Auditory	47.0%	35.8%	6.0%	47.8%
Interoceptive	38.3%	26.4%	4.7%	34.8%
Olfactory	34.5%	29.5%	2.7%	26.1%
Proprioceptive	29.6%	28.0%	3.4%	39.1%
Gustation	29.4%	24.4%	2.0%	30.4%
Visual	23.5%	17.6%	1.3%	13%
Vestibular	22.1%	18.7%	2.0%	17.4%

Table S5.

Repeated analysis of the Anxiety (ASC-ASD-P) and Extreme Demand Avoidance (EDA-8) total scores using the Lifelong EHE age-matched groups. The table displays the medians, Interquartile Ranges (IQR), and Kruskal-Wallis tests and subsequent pairwise comparisons. * Significance values have been adjusted by the Bonferroni correction for multiple tests

Measure	Current SD		Past SD		No SD		Lifelong EHE		*H*(3)	*p*	Significant group differences*
	Median	*IQR*	*Median*	*IQR*	*Median*	*IQR*	*Median*	*IQR*			
ASC-ASD-P Total	41	23.5	30.5	18.8	12	9	16	22	61.25	<.001	Current SD = Past SD > No SD = Lifelong EHE
EDA-8 Total	14	10.8	9	10	2	5	7	8	45.68	<.001	Current SD = Past SD > No SD, Past SD = Lifelong EHE, Current SD > Lifelong EHE, Lifelong EHE > No SD
